# Supplementary material for: Smoking as a risk factor for lower extremity peripheral artery disease in women compared to men: A systematic review and meta-analysis
Source: PLoS One. 2024 Apr 24;19(4):e0300963. doi: 10.1371/journal.pone.0300963 (PMC11042699; doi:10.1371/journal.pone.0300963)
Supplement: S1 Table — (PDF) [file pone.0300963.s002.pdf]

S1 Table Description of search strategy and results (3 March 2024, n=4,672 after removing duplicates)

Database	Search strategy	Number of reports
**PubMed**	#1. “Atherosclerosis”[Mesh] OR “Peripheral Vascular Diseases”[Mesh:NoExp] OR “Ankle Brachial Index”[Mesh] OR “ankle-brachial index”[tiab] OR “peripheral arterial disease*”[tiab] OR “peripheral artery disease*”[tiab] OR “arterial occlusive disease*”[tiab] OR “peripheral vascular disease*”[tiab] OR claudication[tiab] OR “acute limb ischemia”[tiab] OR “acute limb ischaemia”[tiab] OR “critical limb ischemia”[tiab] OR “critical limb ischaemia”[tiab] OR “chronic limb threatening ischemia”[tiab] OR “chronic limb threatening ischaemia”[tiab]	105,980
	#2. “sex characteristics”[Mesh] OR “gender identity”[Mesh] OR “sex distribution”[Mesh] OR “sex”[Mesh] OR “Sex Factors”[Mesh] OR sex[tiab] OR gender[tiab] OR (male*[tiab] AND female*[tiab]) OR (men[tiab] AND women[tiab])	1,837,329
	#3. “smoking”[Mesh] OR “Smoking Reduction”[Mesh] OR “Tobacco Smoke Pollution”[Mesh] OR “smoking”[tiab] OR “cigarette*”[tiab] OR “tobacco” [tiab] OR “nicotine” [tiab] OR “smoker*”[tiab]	449,904
	#4. #1 AND #2 AND #3	3,179
**EMBASE** via Ovid (including conference abstracts, Embase, Medline, and Preprints)	1. atherosclerosis/ OR ankle brachial index/ OR peripheral vascular disease/ OR exp peripheral occlusive artery disease/ OR (ankle-brachial-index OR peripheral-arterial-disease* OR peripheral-artery-disease* OR arterial-occlusive-disease* OR peripheral-vascular-disease* OR claudication OR acute-limb-isch?emia OR critical-limb-isch?emia OR chronic-limb-threatening-isch?emia).tw.	267,991
	2. exp sex difference/ OR sex ratio/ OR exp sex/ OR "gender and sex"/ OR gender/ OR gender identity/ OR groups by sex/ OR (sex OR gender OR (male* AND female*) OR (men AND women)).tw.	2,941,735
	3. exp smoking/ OR smoking cessation/ OR smoking cessation program/ OR smoking habit/ OR smoking reduction/ OR (smoking OR cigarette* OR tobacco OR nicotine OR smoker*).tw.	699,604
	4. 1 AND 2 AND 3	10,808
	5. limit 4 to human and embase and exclude medline journals	1,193
**CINAHL** via EBSCOhost	S1. (MH "Atherosclerosis") OR (MH "Peripheral Vascular Diseases") OR (MH "Chronic Limb-Threatening Ischemia") OR (MH "Ankle Brachial Index") OR TI (“ankle brachial index” OR “peripheral arterial disease*” OR “peripheral artery disease*” OR “arterial occlusive disease*” OR “peripheral vascular disease*” OR “claudication” OR “acute limb ischemia” OR “acute limb ischaemia” OR “critical limb ischemia” OR “critical limb ischaemia” OR “chronic limb threatening ischemia” OR “chronic limb threatening ischaemia”) OR AB (“ankle brachial index” OR “peripheral arterial disease*” OR “peripheral artery disease*” OR “arterial occlusive disease*” OR	26,303

Database	Search strategy	Number of reports
	"peripheral vascular disease*" OR "claudication" OR "acute limb ischemia" OR "acute limb ischaemia" OR "critical limb ischemia" OR "critical limb ischaemia" OR "chronic limb threatening ischemia" OR "chronic limb threatening ischaemia")	
	S2. (MH "Sex Factors") OR (MH "Gender Identity") OR (MH "Gender Role") OR TI (sex OR gender OR (male* AND female*) OR (men AND women)) OR AB (sex OR gender OR (male* AND female*) OR (men AND women))	516,864
	S3. (MH "Smoking+") OR (MH "Smoking Cessation Programs") OR (MH "Smoking Cessation Assistance (Iowa NIC)") OR (MH "Tobacco Use Cessation Products+") OR TI (smoking OR cigarette* OR tobacco OR nicotine OR smoker*) OR AB (smoking OR cigarette* OR tobacco OR nicotine OR smoker*)	145,081
	S4. S1 AND S2 AND S3	799
