# Supplementary material for: Smoking as a risk factor for lower extremity peripheral artery disease in women compared to men: A systematic review and meta-analysis
Source: PLoS One. 2024 Apr 24;19(4):e0300963. doi: 10.1371/journal.pone.0300963 (PMC11042699; doi:10.1371/journal.pone.0300963)
Supplement: S2 Table — a. Characteristics of included studies (recruitment, study population and design, inclusion and exclusion criteria, and age of participants). AAA denotes abdominal aorta aneurysm, ABI ankle brachial index, CRP C-reactive protein, CVD cardiovascular disease, IC intermittent claudication, NSW New South Wales, PAD peripheral artery disease, SD standard deviation, USA United States of America a. Study base: C community-based, H hospital-based, P population-based b. Study design: C cohort, X cross-sectional. Please refer to S3 File for the refences of studies. b. Characteristics of included studies (smoking and peripheral artery disease identification). ABI denotes ankle brachial index, CVD cardiovascular disease, IC intermittent claudication, ICD International Classification of Diseases, ICD-10-AM International Statistical Classification of Diseases and Related Health Problems, Tenth Revision, Australian Modification, OPCS 4 Classification of Interventions and Procedures, PAD peripheral artery disease, SBP systolic blood pressure, WHO World Health Organization. Please refer to S3 File for the refences of studies. c. Characteristics of included studies (sample sizes). PAD denotes peripheral artery disease. Please refer to S3 File for the refences of studies. d. Characteristics of included studies (sample sizes by PAD status and sex). PAD denotes peripheral artery disease. Please refer to S3 File for the refences of studies. e. Characteristics of included studies (sample sizes by smoking status and sex). PAD denotes peripheral artery disease. Please refer to S3 File for the refences of studies. (PDF) [file pone.0300963.s003.pdf]

S2a Table Characteristics of included studies (recruitment, study population and design, inclusion and exclusion criteria, and age of participants)

**Country year, last name of the first author, study name if available (a,b)**	**Recruitment site and period**	**Study population and inclusion and exclusion criteria**	**Age, years old, mean±SD or as specified**
Australia 2019, Banks[1], 45 and Up Study (P, C)	Medicare Australia enrolment database, January 2006 to December 2009	General population. Aged ≥45, randomly sampled from the general population of NSW. Excluded those with a history of CVD or cancer at baseline.	45 to 64 n=133693, 65 to 79 n=43455, ≥80 n=11019
China 2006, He[2] (P, X)	In an urban area, Wanshoulu Community of Haidian District, Beijing, China, April 2001 to March 2002	General population. Aged ≥60. Excluded those with a large left-right difference (a sign of possible vascular disease in the brachial tree) and with an ABI >1.5.	68.5±5.4, PAD 69.2±6.7, non-PAD 67.3±5.7
China 2008, Zheng[3] (H, X)	Inpatients` or outpatients` clinic at 32 university hospitals in Beijing and Shanghai, July to November 2004	People with hypertension, aged ≥40. Excluded individuals with renal failure, valvular heart disease, pregnancy or current lactation, mental disorder, type 1 diabetes or secondary hypertension, severe debilitating chronic illness (cancer, renal, or hepatic diseases), and serious heart failure in whom ABI could not be measured. Excluded 103 participants with ABI >1.4.	68.17±10.68
China 2023, Yi[4] (H, X)	Department of Cardiology at Beijing Anzhen Hospital between 2018 and 2020	People undergoing coronary artery bypass grafting. Excluded patients with incomplete or missing data.	61.62±8.52
England 2015, Pujades-Rodriguez[5], Cardiovascular disease research using Linked Bespoke studies	Patient data linked across four data sources: the Clinical Practice Research Datalink, the Myocardial Ischemia National Audit Project registry,	General population. Aged ≥30, registered in a practice meeting research data recording standard for ≥1 year. Exclude patients with missing record of	Women 47.9±16.0, men 46.0±14.3

Country year, last name of the first author, study name if available (a,b)	Recruitment site and period	Study population and inclusion and exclusion criteria	Age, years old, mean±SD or as specified
and Electronic Records (CALIBER) (P, C)	Hospital Episodes Statistics, and the Office of National Statistics, January 1997 to March 2010	sex, those with a history of CVD and those pregnant within 6 months of the eligibility date.	
Finland 2016, Heikkilä[6], The Harmonica project (C, X)	Residents of the rural town of Harjavalta in Finland, 2005 to 2006	General population. Included those aged 45 to 70 years who had one or more CVD risk factor(s): waist circumference (cm) ≥80 (women) or 94 (men), latest measured blood pressure of ≥140/90 mmHg, history of gestational diabetes or hypertension, and family history of premature CVD. ABI was measured from high cardiovascular-risk subjects with hypertension, metabolic syndrome, pre-diabetes, diabetes, body mass index ≥30 kg/m^2 or a 10-year risk of cardiovascular death of ≥5% according to the Systematic Coronary Risk Evaluation system. Excluded those with previously diagnosed diabetes, CVD, renal disease, or intermittent claudication from the analyses.	Women 58.8±6.9<br>Men 58.1±6.7
Norway 2005, Jensen[7],<br>The Nord-Trøndelag Health Study (Helseundersøkelsen i Nord-Trøndelag: HUNT 2) (P, X)	Nord-Trøndelag County, August 1995 to June 1997	General population. Residents aged 40 to 69 in the analyses.	Women with IC 58.6±8.1, women without IC 52.6±8.5, men with IC 59.1±8.3, men without IC 52.9±8.5
Scotland 2017, Tunstall-Pedoe[8], Scottish Heart Health Extended Cohort (P, C)	1) 23 districts of Scotland, 1984 to 1987;	General population. 1) The Scottish Heart Health Study, aged 40 to 59, randomly recruited across 23 districts of Scotland; 2) Scottish MONICA aged 25	49.0±8.3, women 49.0±8.3, men 49.0±8.3

Country year, last name of the first author, study name if available (a,b)	Recruitment site and period	Study population and inclusion and exclusion criteria	Age, years old, mean±SD or as specified
	2) Edinburgh and North Glasgow, 1986; and 3) North Glasgow, 1989, 1992, and 1995	to 64 from Edinburgh and North Glasgow; and 3) North Glasgow aged 25 to 75. Excluded those with CVD at baseline.	
Spain 2009, Ramos[9] (P, X)	A random population sample of participants from the city of Girona (around 70,000 inhabitants) and two rural towns, stratified by age and sex, 2005 to 2006	General population. Aged 35 to 79. Excluded ABI >1.39 from the analyses.	Women 55.9±12.3, men 56.6±12.3
Spain 2010, Alzamora[10], the Peripheral Arterial disease study (P, X)	28 primary healthcare centers within the metropolitan area of the city of Barcelona and the county of Barcelonès Nord-Maresme, including urban and semi-rural centers, September 2006 to June 2008	General population. Randomly selected (simple random sampling) from a database, aged >49. Excluded ABI >1.4 from analyses.	64.9±8.9, PAD 70.70±9.26, non-PAD 64.22±8.60
Spain 2021, Gonçalves-Martins[11] (P, X)	Health-integrated area AIS-Barcelona Nord, with a population base of 400,000, November 2017 to December 2018	General population. Noninstitutionalized health care cardholders of 65 years old, resided in the area for ≥6 months. As part of a larger pilot screening program evaluating AAA, this study excluded those with a previous AAA diagnosis or repaired, severe comorbidities that may contraindicate AAA elective repair. Excluded those with incorrect postal data, terminal disease, those who did not show up for the scheduled appointment after 3 occasions, those who did not wish to participate or did not respond to invitation	all participants were 65

Country year, last name of the first author, study name if available (a,b)	Recruitment site and period	Study population and inclusion and exclusion criteria	Age, years old, mean±SD or as specified
		letters. 18 participants with ABI >1.4 were included in analyses: 16 grouped as non-PAD and 2 as PAD due to contralateral ABI <0.9.	
Spain 2023, Bermúdez-López[12], The ILERVAS study (C, X)	32 primary basic health areas of the province of Lleida, Spain, January 2015 to December 2018	Population with low-to-moderate cardiovascular risk. Included women aged 50 to 70 years and men aged 45 to 65 years with 1 or more of: hypertension, dyslipidemia, body mass index ≥30, smoking, and a first-degree relative who developed premature CVD (55 years for men and 65 years for women). Excluded those with a clinical history of diabetes, chronic kidney disease, angina, myocardial infarction, stroke, PAD, intestinal or other ischemia, history of arterial surgery, active neoplasia, life expectancy <18 months, or long-term home care or institutionalized population.	Women<br>Non-smoker: 62.02±5.74<br>Former smoker: 57.74±5.31<br>Current smoker: 56.43±4.66<br><br>Men<br>Non-smoker: 55.10±6.19<br>Former smoker: 55.99±5.80<br>Current smoker: 53.98±5.61
UK 2023, Xu[13], UK Biobank Study (P, C)	22 assessment centers across the UK, 2006 and 2010	General population. Aged 40 to 69. Excluded those with PAD hospitalization before baseline.	Women 56.3±8.0, men 56.7 ±8.2
USA 2000, Ness[14] (C, X)	A primary care outpatient geriatrics practice of the Department of Geriatrics and Adult Development at Mount Sinai Medical Centre, 1998 to 1999	General population.	Women 81±8, men 80±8

Country year, last name of the first author, study name if available (a,b)	Recruitment site and period	Study population and inclusion and exclusion criteria	Age, years old, mean±SD or as specified
USA 2002, Lamar Welch[15], the Inter-Tribal Heart Project (C, X)	Indian Health Services clinics in the Red Lake and White Earth reservations in Minnesota, and the Menominee reservation in Wisconsin. August 1992 to July 1994	General population. An age-stratified random sample of active users of the Indian Health Services clinics at each reservation, aged ≥25 years old.	47.6, range 25 to 92
USA 2005, Zheng[16], the Atherosclerosis Risk in Communities study (P, X)	Four communities: Forsyth County NC, Jackson MS, suburban Minneapolis MN, and Washington County MD, 1987 to 1989	General population. Selected by a probability sample of eligible adults aged 45 to 64 to provide a total of 15,792 men and women. Excluded 47 individuals who were not African American or White, and 572 individuals with missing ABI measurements in the current study.	African American: women 53±6, men 54±6; White: women 54±6, men 55±6
USA 2014, Hiramoto[17], The Life Line Screening program (C, X)	20,000 sites across the USA, April 2005 to August 2011, community-based screening services to help make people aware of unrecognized health problems	General population. Voluntary screening population of individuals who were willing to self-pay for diagnostic tests. Included participants with measurement of both ABI and CRP, and complete information on hypercholesterolemia, hypertension, and smoking. Any participant who reported a previous procedure to treat lower-extremity PAD was excluded.	Women 62.1±10.6, men 60.9±10.7

AAA denotes abdominal aorta aneurysm, ABI ankle brachial index, CRP C-reactive protein, CVD cardiovascular disease, IC intermittent claudication, NSW New South Wales, PAD peripheral artery disease, SD standard deviation, USA United States of America

a. Study base: C community-based, H hospital-based, P population-based
b. Study design: C cohort, X cross-sectional

Please refer to S3 File for the refences of studies.

S2b Table Characteristics of included studies (smoking and peripheral artery disease identification)

**Study**	**Method of data collection on smoking status and related variables including levels of smoking status**	**PAD definitions, screening and/or diagnostic methods and/or process**
Australia 2019, Banks[1]	Questionnaire and linked Admitted Patient Data Collection data. Never smokers were participants who answered “No” to the first question, “Have you ever been a regular smoker?” Current smokers were those who answered “Yes” to the first question and “Yes” to the question about being a smoker now, and past smokers were those who indicated that they had ever been a regular smoker and that they were not a regular smoker now.	ICD-10-AM I70-I74. Diagnosis code fields in the hospital data and the underlying cause of death codes in the death data.
China 2006, He[2]	Never smoked, former smoker, and current smoker. An ever-smoker was defined as one who had smoked at least one cigarette daily for ≥1 year. Current smokers were ever-smokers who were still smoking at the time of the interview, and former smokers were those who had stopped for ≥2 years. Pack-years of smoking were calculated by multiplying the average number of cigarettes smoked daily by the number of years of smoking and dividing by 20.	IC according to the WHO/Rose questionnaire or ABI <0.9. The ratio of SBP at the ankle (Doppler, at dorsal pedal or posterior artery) to the SBP at the arm (mercury sphygmomanometer) was calculated for each leg. A lower ABI value was adopted. Measurement of all subjects was done by a trained research nurse and a physician. The quality control procedures showed good agreement in ABI between the two observers and two measurements 20 days apart.
China 2008, Zheng[3]	Questionnaire. Current versus non-current. Smoking status was determined using answers to the question: “Do you now smoke cigarettes?”	ABI ≤0.90 in either leg. A standardized Doppler ultrasonic device was used to measure SBP at bilateral brachial and posterior tibial arteries in the supine position after a 5-min rest with the upper body as flat as possible. The ABI for each leg was calculated as the SBP in the posterior tibial artery divided by the higher value of the two arm SBPs. Investigators were specifically trained to perform ABI measurements.
China 2023, Yi [4]	History of smoking.	Each participant received a lower extremity artery sonographic examination by certified experienced ultrasound clinicians. Four arterial

Study	Method of data collection on smoking status and related variables including levels of smoking status	PAD definitions, screening and/or diagnostic methods and/or process
		beds were imaged: femoral, popliteal, and anterior and posterior tibia arteries. Stenosis (mild 40-49%, moderate 50-69%, or severe 70-99%) or occlusion in any of these arteries were defined as PAD.
England 2015, Pujades-Rodriguez[5]	Patient self-reported smoking status was prospectively collected and coded by general practitioners or practice nurses on the date of consultation in the Clinical Practice Research Datalink. The most recent smoking record before study entry was used. Never, former, and current. Those identified as current smokers with no smoking record within the 3 years before study entry were reclassified as having missed smoking data.	ICD 9, OPCS, ICD 10 (search for "PH236" on https://phenotypes.healthdatagateway.org/). PAD as the initial presentations of a CVD event. Peripheral vascular disease, or leg or aortic embolism or thrombosis recorded as a diagnosis during a primary care consultation, as the primary diagnosis during a hospitalization, or as the underlying cause of death. PAD procedures (excluding repair of AAA), abnormal PAD ultrasound, Doppler, or angiography in a primary care consultation. PAD procedures during a hospitalization.
Finland 2016, Heikkilä[6]	Current smoking	ABI ≤0.90. ABI measurement was performed once by a single doctor and determined from the SBP in all four limbs with the patient in the supine position. SBP in the brachial artery was measured in both upper arms using an appropriately sized blood pressure cuff and a Doppler instrument in the antecubital fossa. SBP was measured from both lower limbs, placing the cuff just above the malleoli using the dorsal pedis artery. If the dorsalis pedis pulse was not detected, the posterior tibial artery pulse was used. ABI was calculated by dividing the lower ankle SBP with the higher brachial SBP.
Norway 2005, Jensen[7]	Questionnaire self-filled in at home. Individuals who had never smoked cigarettes daily were considered as never smokers and those who reported previous or current daily smoking were classified as former or current smokers, respectively. Smokers were asked to report how many cigarettes they currently smoked per day or how many cigarettes per day they previously used	Norwegian translation of the Edinburgh Claudication Questionnaire: four simple questions suitable for self-administration. People with IC gave a history of: (1) pain in one or both legs during walking; (2) pain located in the calf; (3) pain disappears when stops walking for a while; and (4) pain absent at rest.

Study	Method of data collection on smoking status and related variables including levels of smoking status	PAD definitions, screening and/or diagnostic methods and/or process
	to smoke. Smoking 20 cigarettes daily for 1 year equals one pack year. Age of starting smoking was collected among current smokers.	
Scotland 2017, Tunstall-Pedoe[8]	A detailed questionnaire on smoking and measurement of expired air carbon monoxide, serum thiocyanate, and serum cotinine. Current, non-current.	ICD 9 440.2, 443.9, 250.6; ICD 10 I70.2, I73.9, E10.5, E11.5, E12.5, E13.5, E14.5. Mortality and hospital inpatient episodes were followed by periodic national record linkage.
Spain 2009, Ramos[9]	Standardized questionnaire. Smokers (current or quit <1 year), former smokers (quit ≥1 year) or never smokers. Former smokers and never smokers were regarded as non-current smokers.	ABI<0.9 regardless of IC (measured on the Edinburgh questionnaire) was used to define PAD. SBP was measured in the brachial artery in the antecubital fossa in both arms, with a continuous Doppler device. The cuff was then applied to the distal calf, and the Doppler probe was used to determine SBP in supine position at the right and left posterior and anterior tibial arteries. Right and left ABI were calculated as the ratio of the highest of the two SBP in lower limbs (posterior and anterior tibial arteries) to the average of the right and left brachial SBP, unless there was a discrepancy ≥10 mmHg between the two arms (in which case the highest reading was used). The lower of the two ABI values obtained from the left and the right ankle was used for analysis. Operators were meticulously trained by a senior vascular surgeon. A protocol of independent measurements assessed operator performance and found low inter- and intra-operator variability, showing an intraclass correlation coefficient of 0.92 and 0.94, respectively.
Spain 2010, Alzamora[10]	Never, former, and current.	Defined as ABI <0.9. Examined by two trained healthcare professionals under standardized conditions, using standardized Doppler Ultrasonic device. Two paramaleolar arteries of both lower extremities were examined. For each leg, the higher value of the two SBP (tibial posterior and anterior artery) was used. The SBP of the higher arm was used. If the ABI was <0.9, the technique was performed by the other

Study	Method of data collection on smoking status and related variables including levels of smoking status	PAD definitions, screening and/or diagnostic methods and/or process
		professional. If the second professional found an ankle arm index ≥0.9 the first repeated the test and the latter value was recorded as the result.
Spain 2021, Gonçalves-Martins[11]	Face-to-face interview. Active, former (more than a year without smoking), or nonsmoker.	ABI <0.9 in one or both lower extremities. Measured according to the American Heart Association statement: after a 5-min rest, with the subjects in a supine position, SBP was measured at the level of the posterior and anterior tibial arteries of both lower extremities and at the level of the brachial artery of both upper extremities, using a continuous wave Doppler probe. Calculated through the ratio of SBP of the tibial arteries to the highest brachial SBP.
Spain 2023, Bermúdez-López[12]	Smoking status was evaluated in the mobile unit through self-report. Primary variables were smoking habit (non-smoker, former, current), and smoking burden (tobacco packs-year). Former smokers were participants with history of smoking who had not smoked cigarettes within the previous 30 days. Current smokers were participants who consumed cigarettes within the previous 30 days. Pack-years of smoking were determined in current and former smokers and computed as the average number of packs of cigarettes per day multiplied by the duration of smoking in years. Pack-years of smoking were stratified in terciles as low (≤13.53), medium (13.54-29.3), and high (>29.3).	Arterial ultrasound was performed in femoral (common and superficial) arteries. According to Mannheim consensus, an atheroma plaque was defined as a focal encroachment into the lumen of the artery ≥1.5 mm.
UK 2023, Xu[13]	Touchscreen questionnaires and nurse-led interviews. Self-reported as never, former, or current smokers. Current smokers` daily consumption of cigarettes: 1-9, 10-19 and ≥20 cigarettes.	ICD-9 440.2-4, 440.8-9, 443.9, 250.7; ICD-10 I70.0, I70.2, I70.8-9, I73.9, I79.2, E11.5; OPCS L22.2, L26.1-2, L37, L37.1, L37.3-4, L37.8-9, L38.1, L38.8-9, L39.1, L39.5, L39.8-9, L50 (L50.1-6, L50.8-9), L51 (L51.1-6, L51.8-9), L52, L52.1-2, L52.8-9, L53.8-9, L54.1, L54.4, L54.8-9, L58

Study	Method of data collection on smoking status and related variables including levels of smoking status	PAD definitions, screening and/or diagnostic methods and/or process
		(L58.1-9), L59 (L59.1-9), L60, L60.1-4, L60.8-9, L62.1, L62.8-9, L63.1, L63.5, L63.8-9, L65.2-3, L65.8-9, L66, L66.1, L66.2, L66.5, L66.7, L66.8-9, L68, L68.1-2, L68.3-4, L68.8-9, L71.1, L71.5-7, L71.8-9, L76, L76.1-9, L89 (L89.1-6, L89.8-9). Hospital admission data and the national death register (primary or secondary cause of death).
USA 2000, Ness[14]	Current smoking recorded on charts.	Symptomatic PAD was diagnosed if the person had a documented surgery for PAD or if the person had ischemic pain at rest, ulceration or gangrene in an extremity, intermittent claudication, numbness, coldness, cyanosis, or pallor in an extremity, or trophic changes with dry, scaly, and shiny atrophic skin, diminished hair growth, thickened, brittle toenails or subcutaneous atrophy in an extremity associated with absent or weak arterial pulses.
USA 2002, Lamar Welch[15]	Current, former, or never. Both current and former smokers had smoked ≥100 cigarettes in their lifetime.	ABI <0.9. Two readings were obtained of SBPs in the right arm and right and left ankles using an Imex Mascot Doppler, with the participant supine on the examination table. Two ABIs were calculated by dividing the mean right and left ankle SBP, respectively, by the mean SBP in the right arm. The smaller of the two ABIs was adopted.
USA 2005, Zheng[16]	"Current, past, or nonsmoker"	ABI ≤0.9. Resting ankle and brachial SBPs were measured by trained and certified sonographers in each study center using a Dinamap 1846SX automated oscillometer device (Critikon, Inc., Tampa FL). Selection of the appropriate cuff size and cuffing technique were carefully implemented in accordance with the study protocol. The ankle measurements were taken on one randomly selected leg in the prone position using the "contour" method of wrapping. The posterior tibial artery was used as the location for the marker line on the cuff for the "over the artery position". Two readings of ankle SBP were taken 5 to 8 minutes apart and brachial SBP was taken in the supine

Study	Method of data collection on smoking status and related variables including levels of smoking status	PAD definitions, screening and/or diagnostic methods and/or process
		position automatically every 5 minutes. For most participants, two ankle SBP readings and at least two brachial SBP readings were taken. The correlation between duplicate measurements was 0.92 in the leg, and 0.90 in the arm. The ABI was calculated as the average of the two ankle SBP readings divided by the average of the first two brachial SBP readings.
USA 2014, Hiramoto[17]	Current, non-current.	ABI ≤0.90 versus ABI 1.01 to 1.29. SBP were measured in both arms (bilateral brachial arteries) and both ankles (bilateral posterior tibial and dorsalis pedis arteries). The left ABI was calculated by dividing the highest of the left posterior tibial or dorsalis pedis SBP by the highest of the brachial pressures. Similarly, the right ABI was calculated by dividing the highest of the right posterior tibial or dorsalis pedis SBP by the highest of the brachial pressures. The lower value between the 2 legs was adopted. For those who underwent multiple screenings, only the first screening was used.

ABI denotes ankle brachial index, CVD cardiovascular disease, IC intermittent claudication, ICD International Classification of Diseases, ICD-10-AM International Statistical Classification of Diseases and Related Health Problems, Tenth Revision, Australian Modification, OPCS 4 Classification of Interventions and Procedures, PAD peripheral artery disease, SBP systolic blood pressure, WHO World Health Organization.

Please refer to S3 File for the refences of studies.

S2c Table Characteristics of included studies (sample sizes)

Study [Ref]	Whole sample	By PAD		By sex	
		Yes	No	Women	Men
Australia 2019, Banks[1]	188167	2311	185856	105375	82792
China 2006, He[2]	2334	462	1872	1390	944
China 2008, Zheng[3]	5186	1374	3812	2437	2749
China 2023, Yi[4]	1001	470	531	232	769
England 2015, Pujades-Rodriguez[5]	1413749	11519	1402230	766349	647400
Finland 2016, Heikkilä[6]	972	49	923	517	455
Norway 2005, Jensen[7]	19748	230	19518	10343	9405
Scotland 2017, Tunstall-Pedoe[8]	15737	499	15238	8185	7552
Spain 2009, Ramos[9]	6172	277	5895	3269	2903
Spain 2010, Alzamora[10]	3551	286	3265	1954	1597
Spain 2021, Gonçalves-Martins[11]	1174	73	1101	479	695
Spain 2023, Bermúdez-López[12]	8330	4600	3730	4222	4108
UK 2023, Xu[13]	500207	7660	492547	272704	227503
USA 2000, Ness[14]	1911	284	1627	1444	467
USA 2002, Lamar Welch[15]	1333	85	1248	842	491
USA 2005, Zheng[16]	15173	463	14710	8378	6795
USA 2014, Hiramoto[17]	163551	7343	156208	102455	61096

PAD denotes peripheral artery disease.
Please refer to S3 File for the refences of studies.

S2d Table Characteristics of included studies (sample sizes by PAD status and sex)

**Study [Ref]**	Whole sample	By PAD					
		Whole sample		Women		Men	
		Yes	No	Yes	No	Yes	No
Australia 2019, Banks[1]	188167	2311	185856	895	104480	1416	81376
China 2006, He[2]	2334	462	1872	322	1068	140	804
China 2008, Zheng[3]	5186	1374	3812	687	1750	687	2062
China 2023, Yi[4]	1001	470	531	94	138	376	393
England 2015, Pujades-Rodriguez[5]	1413749	11519	1402230	5351	760998	6168	641232
Finland 2016, Heikkilä[6]	972	49	923	26	491	23	432
Norway 2005, Jensen[7]	19748	230	19518	122	10221	108	9297
Scotland 2017, Tunstall-Pedoe[8]	15737	499	15238	208	7977	291	7261
Spain 2009, Ramos[9]	6172	277	5895	127	3142	150	2753
Spain 2010, Alzamora[10]	3551	286	3265	108	1846	178	1419
Spain 2021, Gonçalves-Martins[11]	1174	73	1101	18	461	55	640
Spain 2023, Bermúdez-López[12]	8330	4600	3730	1853	2369	2747	1361
UK 2023, Xu[13]	500207	7660	492547	2658	270046	5002	222501
USA 2000, Ness[14]	1911	284	1627	191	1253	93	374
USA 2002, Lamar Welch[15]	1333	85	1248	51	791	34	457
USA 2005, Zheng[16]	15173	463	14710	311	8067	152	6643
USA 2014, Hiramoto[17]	163551	7343	156208	5442	97013	1901	59195

PAD denotes peripheral artery disease.
Please refer to S3 File for the refences of studies.

S2e Table Characteristics of included studies (sample sizes by smoking status and sex)

**Study [Ref]**	Whole sample	By smoking status								
		Whole sample			Women			Men		
		Former	Current	Never	Former	Current	Never	Former	Current	Never
Australia 2019, Banks[1]	188167	63427	14711	110029	29879	7591	67905	33548	7120	42124
China 2006, He[2]	2334	375	353	1606	62	120	1209	313	233	397
China 2008, Zheng[3]	5186		2069			241			1828	
China 2023, Yi[4]	1001			502						
England 2015, Pujades-Rodriguez[5]	1413749	229177	287130	897442	112547	134056	519746	116630	153074	377696
Finland 2016, Heikkilä[6]	972		170			61			109	
Norway 2005, Jensen[7]	19748	6117	6070	7342	2651	3312	4265	3466	2758	3077
Scotland 2017, Tunstall-Pedoe[8]	15737		7187			3897			3290	
Spain 2009, Ramos[9]	6172	1615	1380	3177	1197	860	846	418	520	2331
Spain 2010, Alzamora[10]	3551	952	624	1975						
Spain 2021, Gonçalves-Martins[11]	1174	430	174	570	102	50	327	328	124	243
Spain 2023, Bermúdez-López[12]	8330	2546	2496	3288	931	992	2299	1615	1504	989
UK 2023, Xu[13]	500207	171874	52390	273017	85174	24207	161819	86700	28183	111198
USA 2000, Ness[14]	1911		249			155			94	
USA 2002, Lamar Welch[15]	1333		852							
USA 2005, Zheng[16]	15173	4904	3945	6324	1878	2065	4435	3026	1880	1889
USA 2014, Hiramoto[17]	163551									

PAD denotes peripheral artery disease.

Please refer to S3 File for the refences of studies.
