# Supplementary material for: Smoking as a risk factor for lower extremity peripheral artery disease in women compared to men: A systematic review and meta-analysis
Source: PLoS One. 2024 Apr 24;19(4):e0300963. doi: 10.1371/journal.pone.0300963 (PMC11042699; doi:10.1371/journal.pone.0300963)
Supplement: S3 Table — a. Quality assessment of cohort studies using the Newcastle-Ottawa scaleTable e-3b Quality assessment of cross-sectional studies using the Newcastle-Ottawa scale. Please refer to S3 File for the refences of studies. b. Table Associations between other measures of smoking and the risk of peripheral artery disease. BMI denotes body mass index, CHD coronary heart disease, HDL-C high-density lipoprotein cholesterol, HR hazard ratio, OR odds ratio, RHR ratio of hazard ratio, ROR ratio of odds ratio, SBP systolic blood pressure, SES socioeconomic status, TC total cholesterol, T1 the lowest tertial, T2 the second tertial, T3 the highest tertial. 1cut-off for tertials of duration of smoking were 18 years for women and 30 years for men. 2cut-off for tertials of pack-years were 10 and 16 pack-years for women, and 13 and 22 for men. 3cut-off for tertials of pack years were 3.0 and 8.3 pack-years for women, and 6.6 and 15.0 for men. 4cut-off points for tertials of time since quitting were 10 and 20 years for women, and 9 and 20 for men. 5cut-off points for tertials of age when started smoking were 18 and 20 years for women, and 16 and 19 for men. Please refer to S3 File for the refences of studies. (PDF) [file pone.0300963.s004.pdf]

S3a Table Quality assessment of cohort studies using the Newcastle-Ottawa scale

Study	Domains and items								Limitations in quality assessment
	Selection				Comparability	Outcome			
	1	2	3	4	5	6	7	8	
Australia 2019, Banks[1]	★	★		★	★★	★	★	★	Ascertainment of smoking status was based on written self-report/"postal questionnaire".
England 2015, Pujades-Rodriguez[5]	★	★	★	★	★	★	★	★	Socioeconomic status was not adjusted for in the analyses.
Scotland 2017, Tunstall-Pedoe[8]	★	★		★	★★	★	★	★	Smoking status was collected from self-administered health record.
UK 2023, Xu[13]		★	★	★	★★	★	★	★	Participants were more likely to live in less socioeconomically deprived areas, and be healthier, than the general population.

1. Representativeness of the exposed cohort: a) Truly representative★; b) Somewhat representative★; c) Selected group; d) No description of the derivation of the cohort.
2. Selection of the non-exposed cohort: a) Drawn from the same community as the exposed cohort★; b) Drawn from a different source; c) No description of the derivation of the non-exposed cohort.
3. Ascertainment of exposure: a) Secure record (e.g., surgical record)★; b) Structured interview★; c) Written self-report; d) No description; e) Other.
4. Demonstration that outcome of interest was not present at start of study: a) Yes★; b) No.
5. Comparability of cohorts based on the design or analysis controlled for confounders: a) The study controls for socioeconomic status★; b) The study control for any additional factor(s), an additional★; c) Cohorts are not comparable on the basis of the design or analysis controlled for confounders.
6. Assessment of outcome: a) Independent blind assessment★; b) Record linkage★; c) Self-report; d) No description; e) Other.
7. Was follow-up long enough for outcomes to occur a) Yes★; b) No.
8. Adequacy of follow-up of cohorts: a) Complete follow up, all subject accounted for★; b) Subjects lost to follow up unlikely to introduce bias, number lost less than or equal to 20% or description of those lost suggested no different from those followed★; c) Follow up rate less than 80% and no description of those lost.

Please refer to S3 File for the refences of studies.

S3b Table Quality assessment of cross-sectional studies using the Newcastle-Ottawa scale

Study	Domains and items							Limitations in quality assessment
	Selection				Comparability	Outcome		
	1	2	3	4	5	6	7	
China 2006, He[2]			★	★	★★	★★	★	Individuals were recruited from selected streets, so it is uncertain how the streets were selected. Sample size was not justified.
China 2008, Zheng[3]				★	★	★	★	Participants were only recruited from hospitals. Sample size was not justified. There were no comparisons between respondents and non-respondents. Socioeconomic status was not adjusted for in the analyses. Assessment of the outcome may be unblind.
China 2023, Yi[4]	★		★		★	★	★	Sample size was not justified. There were no descriptions of the measurement tool to collect smoking information, so it is unclear how the question was asked. Socioeconomic status was not adjusted for in the analyses. Assessment of the outcome may not be independent and may be unblind.
Finland 2016, Heikkilä[6]	★				★	★	★	Sample size was not justified. There were no comparisons between respondents and non-respondents. There were no descriptions of the measurement tool to collect smoking information, so it is unclear how the question was asked. Socioeconomic status was not adjusted for in the analyses. Assessment of the outcome was not independent and unblind.
Norway 2005, Jensen[7]			★		★	★	★	The average income, the prevalence of higher education, and the prevalence of current smokers are a little lower than the average of Norway. Sample size was not justified. There were no descriptions of the measurement tool to collect smoking information, so it is unclear how the question was asked. Socioeconomic status was not adjusted for in the analyses. Assessment of the outcome was self-reported.
Spain 2009, Ramos[9]	★	★	★	★	★	★	★	Socioeconomic status was not adjusted for in the analyses. Assessment of the outcome may be unblind.
Spain 2010, Alzamora[10]	★			★	★★	★	★	Sample size was not justified. There were no comparisons between respondents and non-respondents. Assessment of the outcome may be unblind.
Spain 2021, Gonçalves-Martins[11]					★	★		Only people aged 65 years old were included. Sample size was not justified. There were no comparisons between respondents and non-respondents. There were no descriptions of the measurement tool to collect smoking information, so it is unclear how the question was asked. Socioeconomic status was not adjusted for in the analyses. Assessment of the outcome may not be independent and may be unblind. Adjustments were different in women and men.
Spain 2023, Bermúdez-López[12]	★			★	★	★	★	Sample size was not justified. There were no comparisons between respondents and non-respondents. Socioeconomic

								status was not adjusted for in the analyses. Assessment of the outcome may not be independent and may be unblind.
USA 2000, Ness[14]	★		★	★	★	★	★	Sample size was not justified. Socioeconomic status was not adjusted for in the analyses. Assessment of the outcome was unblind.
USA 2002, Lamar Welch[15]	★					★	★	Sample size was not justified. There were no comparisons between respondents and non-respondents. There were no descriptions of the measurement tool to collect smoking information, so it is unclear how the question was asked. Only the age of the participants was adjusted for in the analyses. Assessment of the outcome may not be independent and may be unblind.
USA 2005, Zheng[16]	★	★	★		★	★	★	There were no descriptions of the measurement tool to collect smoking information, so it is unclear how the question was asked. Socioeconomic status was not adjusted for in the analyses.
USA 2014, Hiramoto[17]					★	★	★	Voluntary screening population of individuals who were willing to self-pay for diagnostic tests, which may not be generalizable to other populations. Sample size was not justified. There were no comparisons between respondents and non-respondents. There were no descriptions of the measurement tool to collect smoking information, so it is unclear how the question was asked. Socioeconomic status was not adjusted for in the analyses. Assessment of the outcome may be unblind.

1. Representativeness of the sample: a) Truly representative of the average in the target population★; b) Somewhat representative of the average in the target population★; c) Selected group of users; d) No description of the sampling strategy.
2. Sample size: a) Justified and satisfactory★; b) Not justified.
3. Non-respondents: a) Comparability between respondents and non-respondents` characteristics is established, and the response rate is satisfactory★; b) The response rate is unsatisfactory, or the comparability between respondents and non-respondents is unsatisfactory; c) No description of the response rate or the characteristics of the responders and the non-responders.
4. Ascertainment of the exposure: a) Validated measurement tool★★; b) Non-validated measurement tool, but the tool is available or described★; c) No description of the measurement tool.
5. The subjects in different outcome groups are comparable, based on the study design or analysis. Confounding factors are controlled. a) The study controls for the most important factor, i.e., socioeconomic status★; b) The study control for any additional factor(s), an additional★.
6. Assessment of the outcome: a) Independent blind assessment★★; b) Record linkage★★; c) Self report★; d) No description.
7. Statistical test: a) The statistical test used to analyze the data is clearly described and appropriate, and the measurement of the association is presented, including confidence intervals or the probability level★; b) The statistical test is not appropriate, not described, or incomplete.

Please refer to S3 File for the refences of studies.
