# Supplementary material for: Smoking as a risk factor for lower extremity peripheral artery disease in women compared to men: A systematic review and meta-analysis
Source: PLoS One. 2024 Apr 24;19(4):e0300963. doi: 10.1371/journal.pone.0300963 (PMC11042699; doi:10.1371/journal.pone.0300963)
Supplement: S4 Table — (PDF) [file pone.0300963.s005.pdf]

S4 Table Associations between other measures of smoking and the risk of peripheral artery disease

Studies	Measures of smoking	Point estimate (95% confidence interval)			Adjusted variables
		Women	Men	Women-to-men	
**Cohort studies**		HR	HR	RHR	
Scotland 2017, Tunstall-Pedoe[8]	Per 1 cigs/day (never smokers had 0 cigs/day)	2.01 (1.85, 2.19)	1.68 (1.54, 1.82)	1.20 (1.06, 1.35)	age
	Expired carbon monoxide, per 1 ppm	1.56 (1.40, 1.73)	1.33 (1.20, 1.47)	1.17 (1.01, 1.36)	age, family history of CHD, SES, cigs/day, SBP, diabetes, TC, HDL-C
	Thiocyanate, per 1 µmol/L	1.94 (1.62, 2.32)	1.63 (1.38, 1.92)	1.19 (0.93, 1.52)	
	Cotinine, per 1 ng/ml	1.72 (1.48, 1.99)	1.42 (1.22, 1.66)	1.21 (0.98, 1.50)	
**Cross-sectional studies**		OR	OR	ROR	
China 2006, He[2]	Intensity and duration, pack-years				age, marital status, education (years: ≤6, 7-12, ≥13), current vs non-current alcohol drinkers, exercise (hours/day: <1, 1-3, ≥4), BMI, hypertension or diabetes, and family histories of CHD or stroke
	1-20 vs 0	1.13 (0.71, 1.81)	1.06 (0.61, 1.70)	1.07 (0.53, 2.13)	
	21-40 vs 0	1.46 (0.79, 2.69)	1.50 (0.91, 2.48)	0.97 (0.44, 2.15)	
	>40 vs 0	3.54 (1.35, 9.31)	2.28 (1.33, 3.91)	1.55 (0.51, 4.69)	
	Years since quit vs never smoked				
	2-9 years	1.27 (0.59, 2.73)	1.74 (1.01, 2.98)	0.73 (0.29, 1.86)	
	≥10 years	0.93 (0.37, 2.31)	1.18 (0.68, 2.03)	0.79 (0.27, 2.29)	
Norway 2005, Jensen[7]	Current or former smokers` cigs/day				age
	T2 vs T1	1.3 (0.6, 2.4)	1.3 (0.6, 2.7)	1.00 (0.36, 2.78)	
	T3 vs T1	0.8 (0.4, 1.7)	1.7 (0.8, 3.5)	0.47 (0.17, 1.32)	
	Current and former smokers` duration of smoking vs never[1]				
	T1	1.3 (0.7, 2.4)	1.3 (0.6, 2.8)	1.00 (0.37, 2.68)	
	T2	2.3 (1.4, 4.0)	1.9 (1.0, 3.6)	1.21 (0.53, 2.77)	
	T3	2.2 (1.4, 3.5)	4.6 (2.5, 8.4)	0.48 (0.22, 1.02)	
	Current smokers` pack-years[2]				
	T2 vs T1	2.4 (1.1, 5.3)	3.3 (1.2, 8.9)	0.73 (0.20, 2.60)	
	T3 vs T1	1.0 (0.4, 2.3)	3.5 (1.3, 9.3)	0.29 (0.08, 1.07)	
	Former smokers` pack-years[3]				
	T2 vs T1	1.0 (0.4, 2.7)	1.9 (0.5, 7.4)	0.53 (0.10, 2.74)	
	T3 vs T1	1.6 (0.7, 3.9)	5.8 (1.7, 19.5)	0.28 (0.06, 1.23)	

	Years since quitting vs current[4]				
	T1	1.1 (0.6, 2.1)	0.6 (0.4, 1.1)	1.83 (0.82, 4.10)	
	T2	1.0 (0.5, 2.1)	0.5 (0.3, 1.0)	2.00 (0.78, 5.10)	
	T3	0.4 (0.2, 0.8)	0.2 (0.1, 0.5)	2.00 (0.69, 5.78)	
	Current smokers` age of starting smoking[5]				
	T3 vs T1	1.4 (0.6, 3.3)	1.1 (0.6, 2.2)	1.27 (0.44, 3.72)	
	T2 vs T1	0.8 (0.4, 1.8)	0.7 (0.4, 1.4)	1.14 (0.43, 3.04)	
Spain 2023, Bermúdez-López[12]	Former or current smokers` pack-years				age, hypertension, obesity, dyslipidemia, prediabetes or diabetes, Mediterranean diet adherence score, neck perimeter and abdominal obesity
	T1 vs never	1.39 (1.13, 1.70)	1.60 (1.30, 1.98)	0.87 (0.65, 1.16)	
	T2 vs never	3.51 (2.80, 4.42)	4.04 (3.26, 5.03)	0.87 (0.63, 1.19)	
	T3 vs never	7.19 (5.45, 9.56)	8.28 (6.55, 10.52)	0.87 (0.60, 1.25)	

BMI denotes body mass index, CHD coronary heart disease, HDL-C high-density lipoprotein cholesterol, HR hazard ratio, OR odds ratio, RHR ratio of hazard ratio, ROR ratio of odds ratio, SBP systolic blood pressure, SES socioeconomic status, TC total cholesterol, T1 the lowest tertial, T2 the second tertial, T3 the highest tertial.

[1]cut-off for tertials of duration of smoking were 18 years for women and 30 years for men

[2]cut-off for tertials of pack-years were 10 and 16 pack-years for women, and 13 and 22 for men

[3]cut-off for tertials of pack years were 3.0 and 8.3 pack-years for women, and 6.6 and 15.0 for men

[4]cut-off points for tertials of time since quitting were 10 and 20 years for women, and 9 and 20 for men

[5]cut-off points for tertials of age when started smoking were 18 and 20 years for women, and 16 and 19 for men

Please refer to S3 File for the refences of studies.
