# Supplementary material for: Smoking as a risk factor for lower extremity peripheral artery disease in women compared to men: A systematic review and meta-analysis
Source: PLoS One. 2024 Apr 24;19(4):e0300963. doi: 10.1371/journal.pone.0300963 (PMC11042699; doi:10.1371/journal.pone.0300963)
Supplement: S1 File — a. Preferred Reporting Items for Systematic Reviews and Meta-Analyses (PRISMA) 2020 for abstracts checklist. b. Preferred Reporting Items for Systematic Reviews and Meta-Analyses (PRISMA) 2020 Checklist. c. Meta-analysis Of Observational Studies in Epidemiology (MOOSE) Guideline. (PDF) [file pone.0300963.s006.pdf]

S1a File Preferred Reporting Items for Systematic Reviews and Meta-Analyses (PRISMA) 2020 for abstracts checklist

Section and Topic	Item #	Checklist item	Reported (Yes/No)
**TITLE**			
Title	1	Identify the report as a systematic review.	Yes
**BACKGROUND**			
Objectives	2	Provide an explicit statement of the main objective(s) or question(s) the review addresses.	Yes
**METHODS**			
Eligibility criteria	3	Specify the inclusion and exclusion criteria for the review.	Yes
Information sources	4	Specify the information sources (e.g., databases, registers) used to identify studies and the date when each was last searched.	Yes
Risk of bias	5	Specify the methods used to assess risk of bias in the included studies.	Yes
Synthesis of results	6	Specify the methods used to present and synthesize results.	Yes
**RESULTS**			
Included studies	7	Give the total number of included studies and participants and summarize relevant characteristics of studies.	Yes
Synthesis of results	8	Present results for main outcomes, preferably indicating the number of included studies and participants for each. If meta-analysis was done, report the summary estimate and confidence/credible interval. If comparing groups, indicate the direction of the effect (i.e., which group is favored).	Yes
**DISCUSSION**			

Section and Topic	Item #	Checklist item	Reported (Yes/No)
Limitations of evidence	9	Provide a brief summary of the limitations of the evidence included in the review (e.g., study risk of bias, inconsistency and imprecision).	Yes
Interpretation	10	Provide a general interpretation of the results and important implications.	Yes
**OTHER**			
Funding	11	Specify the primary source of funding for the review.	Yes
Registration	12	Provide the register name and registration number.	Yes

S1b File Preferred Reporting Items for Systematic Reviews and Meta-Analyses (PRISMA) 2020 Checklist

Section and Topic	Item #	Checklist item	Location where item is reported
TITLE			
Title	1	Identify the report as a systematic review.	Title, Abstract
ABSTRACT			
Abstract	2	See the PRISMA 2020 for Abstracts checklist.	Abstract
INTRODUCTION			
Rationale	3	Describe the rationale for the review in the context of existing knowledge.	Introduction paragraph
Objectives	4	Provide an explicit statement of the objective(s) or question(s) the review addresses.	Introduction paragraph
METHODS			
Eligibility criteria	5	Specify the inclusion and exclusion criteria for the review and how studies were grouped for the syntheses.	Methods subsections “Inclusion and exclusion criteria”, “Strategy for data synthesis”
Information sources	6	Specify all databases, registers, websites, organizations, reference lists and other sources searched or consulted to identify studies. Specify the date when each source was last searched or consulted.	Methods subsection “Search strategy and screening” Supplementary table e-1 and appendix 2 to 3
Search strategy	7	Present the full search strategies for all databases, registers, and websites, including any filters and limits used.	Supplementary table e-1
Selection process	8	Specify the methods used to decide whether a study met the inclusion criteria of the review, including how many reviewers screened each record and each report retrieved, whether they worked independently, and if applicable, details of automation tools used in the process.	Methods subsection “Search strategy and screening”
Data collection process	9	Specify the methods used to collect data from reports, including how many reviewers collected data from each report, whether they worked independently, any processes for obtaining or confirming data from study investigators, and if applicable, details of automation tools used in the process.	Methods subsection “Data extraction and quality assessment”
Data items	10a	List and define all outcomes for which data were sought. Specify whether all results that were compatible with each outcome domain in each study were sought (e.g., for all	Methods subsection “Inclusion and exclusion criteria”

Section and Topic	Item #	Checklist item	Location where item is reported
		measures, time points, analyses), and if not, the methods used to decide which results to collect.	
	10b	List and define all other variables for which data were sought (e.g., participant and intervention characteristics, funding sources). Describe any assumptions made about any missing or unclear information.	Methods subsection “Data extraction and quality assessment”
Study risk of bias assessment	11	Specify the methods used to assess risk of bias in the included studies, including details of the tool(s) used, how many reviewers assessed each study and whether they worked independently, and if applicable, details of automation tools used in the process.	Methods subsection “Data extraction and quality assessment”
Effect measures	12	Specify for each outcome the effect measure(s) (e.g., risk ratio, mean difference) used in the synthesis or presentation of results.	Methods subsection “Strategy for data synthesis”
Synthesis methods	13a	Describe the processes used to decide which studies were eligible for each synthesis (e.g., tabulating the study intervention characteristics and comparing against the planned groups for each synthesis (item #5)).	Methods subsection “Strategy for data synthesis”
	13b	Describe any methods required to prepare the data for presentation or synthesis, such as handling of missing summary statistics, or data conversions.	Methods subsection “Strategy for data synthesis”, first sentence
	13c	Describe any methods used to tabulate or visually display results of individual studies and syntheses.	Methods subsection “Strategy for data synthesis”, Supplementary tables e-2a to e-2e
	13d	Describe any methods used to synthesize results and provide a rationale for the choice(s). If meta-analysis was performed, describe the model(s), method(s) to identify the presence and extent of statistical heterogeneity, and software package(s) used.	Methods subsection “Strategy for data synthesis”
	13e	Describe any methods used to explore possible causes of heterogeneity among study results (e.g., subgroup analysis, meta-regression).	Methods subsection “Strategy for data synthesis”, but further analyses were not possible as the number of studies in each comparison was small.
	13f	Describe any sensitivity analyses conducted to assess robustness of the synthesized results.	
Reporting bias assessment	14	Describe any methods used to assess risk of bias due to missing results in a synthesis (arising from reporting biases).	

Section and Topic	Item #	Checklist item	Location where item is reported
Certainty assessment	15	Describe any methods used to assess certainty (or confidence) in the body of evidence for an outcome.	Methods subsection “Data extraction and quality assessment”
RESULTS			
Study selection	16a	Describe the results of the search and selection process, from the number of records identified in the search to the number of studies included in the review, ideally using a flow diagram.	Figure 1, Results paragraphs 1 to 3
	16b	Cite studies that might appear to meet the inclusion criteria, but which were excluded, and explain why they were excluded.	Supplementary appendix 2
Study characteristics	17	Cite each included study and present its characteristics.	Results paragraphs 1 to 3, Supplementary tables e-2a to e-2e
Risk of bias in studies	18	Present assessments of risk of bias for each included study.	Results subsections “Quality assessment”, supplementary tables e-3a to e-3b
Results of individual studies	19	For all outcomes, present, for each study: (a) summary statistics for each group (where appropriate) and (b) an effect estimate and its precision (e.g. confidence/credible interval), ideally using structured tables or plots.	Results subsections “Associations reported in cohort studies”, “Associations reported in cross-sectional studies”, Figures 2 to 3
Results of syntheses	20a	For each synthesis, briefly summarize the characteristics and risk of bias among contributing studies.	Supplementary tables e-2a to e-2e, e-3a to e-3b
	20b	Present results of all statistical syntheses conducted. If meta-analysis was done, present for each the summary estimate and its precision (e.g. confidence/credible interval) and measures of statistical heterogeneity. If comparing groups, describe the direction of the effect.	95% confidence intervals are presented throughout. Between-study heterogeneity was examined using I^2 statistic and Cochran’s Q test.
	20c	Present results of all investigations of possible causes of heterogeneity among study results.	Methods subsection “Strategy for data synthesis”, but further analyses were not possible as the number of studies in each comparison was small.
	20d	Present results of all sensitivity analyses conducted to assess the robustness of the synthesized results.	
Reporting biases	21	Present assessments of risk of bias due to missing results (arising from reporting biases) for each synthesis assessed.	
Certainty of evidence	22	Present assessments of certainty (or confidence) in the body of evidence for each outcome assessed.	95% confidence intervals are presented throughout. Between-study heterogeneity

Section and Topic	Item #	Checklist item	Location where item is reported
			was examined using I^2 statistic and Cochran's Q test.
DISCUSSION			
Discussion	23a	Provide a general interpretation of the results in the context of other evidence.	Discussion paragraph 2
	23b	Discuss any limitations of the evidence included in the review.	Discussion subsection "Strengths and limitations"
	23c	Discuss any limitations of the review processes used.	Discussion subsection "Strengths and limitations"
	23d	Discuss implications of the results for practice, policy, and future research.	Discussion subsection "Conclusions"
OTHER INFORMATION			
Registration and protocol	24a	Provide registration information for the review, including register name and registration number, or state that the review was not registered.	PROSPERO (CRD42022352318)
	24b	Indicate where the review protocol can be accessed, or state that a protocol was not prepared.	https://www.crd.york.ac.uk/prospero/display_record.php?RecordID=352318
	24c	Describe and explain any amendments to information provided at registration or in the protocol.	No amendments
Support	25	Describe sources of financial or non-financial support for the review, and the role of the funders or sponsors in the review.	"Funding sources"
Competing interests	26	Declare any competing interests of review authors.	"Declaration of interests"
Availability of data, code, and other materials	27	Report which of the following are publicly available and where they can be found: template data collection forms; data extracted from included studies; data used for all analyses; analytic code; any other materials used in the review.	"Author contributions"

From: Page MJ, McKenzie JE, Bossuyt PM, Boutron I, Hoffmann TC, Mulrow CD, et al. The PRISMA 2020 statement: an updated guideline for reporting systematic reviews. BMJ 2021;372:n71. doi: 10.1136/bmj.n71

For more information, visit: http://www.prisma-statement.org/

S1c File Meta-analysis Of Observational Studies in Epidemiology (MOOSE) Guideline

**Reporting of background should include**	
Problem definition	Introduction paragraph
Hypothesis statement	NA, as the relationship between smoking and peripheral artery disease (PAD) may or may not differ by sex.
Description of study outcome(s)	Introduction paragraph
Type of exposure or intervention used	Introduction paragraph, intervention NA
Type of study designs used	Introduction paragraph, "a systematic review with meta-analyses"
Study population	Introduction paragraph
**Reporting of search strategy should include**	
Qualifications of searchers (e.g., librarians and investigators)	Methods subsection, "Search strategy and screening"
Search strategy, including time period included in the synthesis and keywords	Methods subsection, "Search strategy and screening", Supplementary table e-1
Effort to include all available studies, including contact with authors	Methods subsection, "Search strategy and screening"
Databases and registries searched	Methods subsection, "Search strategy and screening", Supplementary table e-1
Search software used, name and version, including special features used (e.g., explosion)	
Use of hand searching (e.g., reference lists of obtained articles)	
List of citations located and those excluded, including justification	Appendix 2
Method of addressing articles published in languages other than English	In the search, "No language restrictions were applied." All identified records have titles and abstracts in English, and we did not identify any potentially eligible studies that were published in languages other than English.
Method of handling abstracts and unpublished studies	We included "peer-reviewed cohort, case-cohort, and cross-sectional studies". That is, abstracts and unpublished studies were excluded.
Description of any contact with authors	Methods subsection, "Search strategy and screening"
**Reporting of methods should include**	
Description of relevance or appropriateness of studies assembled for assessing the hypothesis to be tested	Methods subsection, "Inclusion and exclusion criteria"
Rationale for the selection and coding of data (e.g., sound clinical principles or convenience)	"pre-specified data collection forms"
Documentation of how data were classified and coded (e.g., multiple raters, blinding, and interrater reliability)	Methods subsection, "Data extraction and quality assessment"
Assessment of confounding (e.g., comparability of cases and controls in studies where appropriate)	

Assessment of study quality, including blinding of quality assessors; stratification or regression on possible predictors of study results	Methods subsections, “Data extraction and quality assessment”, “Strategy for data synthesis”
Assessment of heterogeneity	Methods subsection, “Strategy for data synthesis”
Description of statistical methods (e.g., complete description of fixed or random effects models, justification of whether the chosen models account for predictors of study results, dose-response models, or cumulative meta-analysis) in sufficient detail to be replicated	
Provision of appropriate tables and graphics	Figures 1 to 3, Supplementary tables and appendices
**Reporting of results should include**	
Graphic summarizing individual study estimates and overall estimate	Figures 2 to 3
Table giving descriptive information for each study included	Supplementary tables e-2a to e-2e
Results of sensitivity testing (e.g., subgroup analysis)	Methods subsection “Strategy for data synthesis”, but further analyses were not possible as the number of studies in each comparison was small.
Indication of statistical uncertainty of findings	95% confidence intervals are presented throughout.
**Reporting of discussion should include**	
Quantitative assessment of bias (e.g., publication bias)	Discussion paragraphs 3 to 4
Justification for exclusion (e.g., exclusion of non-English-language citations)	Discussion subsection, “Strengths and limitations”
Assessment of quality of included studies	
**Reporting of conclusions should include**	
Consideration of alternative explanations for observed results	Discussion subsection, “Conclusions”
Generalization of the conclusions (i.e., appropriate for the data presented and within the domain of the literature review)	
Guidelines for future research	
Disclosure of funding source	“Funding sources”
