# Supplementary material for: Smoking as a risk factor for lower extremity peripheral artery disease in women compared to men: A systematic review and meta-analysis
Source: PLoS One. 2024 Apr 24;19(4):e0300963. doi: 10.1371/journal.pone.0300963 (PMC11042699; doi:10.1371/journal.pone.0300963)
Supplement: S2 File — (PDF) [file pone.0300963.s007.pdf]

S2 File List of excluded studies in full-text screening stage with reasons (n=283)

Backward stepwise selection procedure to define two sets of risk factors by sex, and smoking was not retained in both sexes (n=5)

1) Grenon SM, Cohen BE, Smolderen K, Vittinghoff E, Whooley MA, Hiramoto J. Peripheral arterial disease, gender, and depression in the Heart and Soul Study. J Vasc Surg. 2014;60:396-403
2) Guerchet M, Aboyans V, Mbelesso P, Mouanga AM, Salazar J, Bandzouzi B, et al. Epidemiology of peripheral artery disease in elder general population of two cities of Central Africa: Bangui and Brazzaville. Eur J Vasc Endovasc Surg. 2012;44:164-169
3) Jamee A, Aboyans V, Magne J, Elhendy M, Marie Preux P, Lacroix P. Epidemiology of peripheral artery disease in Palestine. Vasa. 2020;49:323-329
4) Tavintharan S, Ning C, Su Chi L, Tay W, Shankar A, Shyong Tai E, et al. Prevalence and risk factors for peripheral artery disease in an Asian population with diabetes mellitus. Diab Vasc Dis Res. 2009;6:80-86
5) Tseng CH. Sex difference in the distribution of atherosclerotic risk factors and their association with peripheral arterial disease in Taiwanese type 2 diabetic patients. Circ J. 2007;71:1131-1136

Estimation of the variance not reported (n=4)

1) Kannel WB, McGee DL. Update on some epidemiologic features of intermittent claudication: the Framingham Study. J Am Geriatr Soc. 1985;33:13-18
2) Kannel WB. Risk factors for atherosclerotic cardiovascular outcomes in different arterial territories. J Cardiovasc Risk. 1994;1:333-339
3) Mellström D, Svanborg A. Tobacco smoking--a major cause of sex differences in health. Compr Gerontol A. 1987;1:34-39
4) Reunanen A, Takkunen H, Aromaa A. Prevalence of intermittent claudication and its effect on mortality. Acta Med Scand. 1982;211:249-256

No reports on the association between smoking and peripheral arterial disease (n=107)

These included studies: 1) with no assessment of smoking; 2) where smoking was a covariate or a stratified variable, when examining other risk factors of interest; 3) where peripheral arterial disease was not the outcome or was a component of a composite outcome; or 4) only recruited participants with peripheral arterial disease.

1) Ali Z, Ellington AA, Mosley TH, Jr., Kullo IJ. Association of serum osteoprotegerin with ankle-brachial index and urine albumin: creatinine ratio in African-Americans and non-Hispanic whites. Atherosclerosis. 2009;206:575-580
2) Ali Z, Sarcia P, Mosley TH, Jr., Kondragunta V, Kullo IJ. Association of serum myeloperoxidase with the ankle-brachial index and peripheral arterial disease. Vasc Med. 2009;14:215-220

3) Al-Mahroos F, Al-Roomi K. Diabetic neuropathy, foot ulceration, peripheral vascular disease and potential risk factors among patients with diabetes in Bahrain: a nationwide primary care diabetes clinic-based study. Ann Saudi Med. 2007;27:25-31
4) Alsadiqi AIM, Subki AH, Abushanab RH, Ocheltree MR, Bajahmom HA, Alsadiqi YIM, et al. Peripheral artery disease risk factors in Jeddah, Saudi Arabia: a retrospective study. Int J Gen Med. 2019;12:49-54
5) Alsuwailem B, Zamzam A, Syed MH, Greco E, Wheatcroft M, de Mestral C, et al. Elevated plasma levels of NT-proBNP in ambulatory patients with peripheral arterial disease. PLoS One. 2021;16:e0253792
6) Al-Zoubi NA, Shatnawi NJ. Gender variation in symptomatic peripheral arterial occlusive disease among type-2 diabetic patients. SAGE Open Med. 2019;7:2050312119840198
7) Aponte J. The prevalence of peripheral arterial disease (PAD) and PAD risk factors among different ethnic groups in the US population. J Vasc Nurs. 2012;30:37-43
8) Baretella O, Buser L, Andres C, Häberli D, Lenz A, Döring Y, et al. Association of sex and cardiovascular risk factors with atherosclerosis distribution pattern in lower extremity peripheral artery disease. Front Cardiovasc Med. 2023;10:1004003.
9) Bergman BP, Mackay DF, Pell JP. Peripheral arterial disease in Scottish military veterans: a retrospective cohort study of 57 000 veterans and 173 000 matched non-veterans. J Public Health (Oxf). 2019;41:e9-e15
10) Bergmark C, Mansoor MA, Swedenborg J, de Faire U, Svardal AM, Ueland PM. Hyperhomocysteinemia in patients operated for lower extremity ischaemia below the age of 50--effect of smoking and extent of disease. Eur J Vasc Surg. 1993;7:391-396
11) Bihari-Varga M, Székely J, Gruber E. Plasma high density lipoproteins in coronary, cerebral and peripheral vascular disease. The influence of various risk factors. Atherosclerosis. 1981;40:337-345
12) Blomster JI, Woodward M, Zoungas S, Hillis GS, Harrap S, Neal B, et al. The harms of smoking and benefits of smoking cessation in women compared with men with type 2 diabetes: an observational analysis of the ADVANCE (Action in Diabetes and Vascular Disease: Preterax and Diamicron modified release Controlled Evaluation) trial. BMJ Open. 2016;6:e009668
13) Bostom AG, Gagnon DR, Cupples LA, Wilson PW, Jenner JL, Ordovas JM, et al. A prospective investigation of elevated lipoprotein (a) detected by electrophoresis and cardiovascular disease in women. The Framingham Heart Study. Circulation. 1994;90:1688-1695
14) Britton KA, Mukamal KJ, Ix JH, Siscovick DS, Newman AB, de Boer IH, et al. Insulin resistance and incident peripheral artery disease in the Cardiovascular Health Study. Vasc Med. 2012;17:85-93
15) Bulpitt CJ, Shipley M, Fletcher AE, Markowe H, Marmot M, Semmence A, et al. Smoking and blood pressure in the leg. J Hypertens. 1993;11:211-215
16) Castleden WM, House AK, Faulkner K, Watt A. Haemoglobin, smoking and peripheral vascular disease. J R Soc Med. 1981;74:586-590

17) Cheng SW, Ting AC, Lau H, Wong J. Epidemiology of atherosclerotic peripheral arterial occlusive disease in Hong Kong. World J Surg. 1999;23:202-206
18) Chung YK, Lee YJ, Kim KW, Cho RK, Chung SM, Moon JS, et al. Serum cystatin C is associated with subclinical atherosclerosis in patients with type 2 diabetes: a retrospective study. Diab Vasc Dis Res. 2018;15:24-30
19) Corlin L, Short MI, Vasan RS, Xanthakis V. Association of the duration of ideal cardiovascular health through adulthood with cardiometabolic outcomes and mortality in the Framingham Offspring Study. JAMA Cardiol. 2020;5:549-556
20) Daher M, Al Rifai M, Mahtta D, Krittanawong C, Berman J, Ullah W, et al. Substance use and premature atherosclerotic cardiovascular disease (from the CDC Behavioral Risk Factor Surveillance System [BRFSS] survey). Am J Cardiol. 2021;152:177-178
21) Daskalopoulou M, George J, Walters K, Osborn DP, Batty GD, Stogiannis D, et al. Depression as a risk factor for the initial presentation of twelve cardiac, cerebrovascular, and peripheral arterial diseases: data linkage study of 1.9 million women and men. PLoS One. 2016;11:e0153838
22) dos Santos VP, Alves CAS, Lopes CF, de Araujo Filho JS. Gender-related differences in critical limb ischemia due to peripheral arterial occlusive disease. J Vasc Bras. 2013;12(4):278-283
23) Erdozain JG, Villar I, Nieto J, Ruiz-Arruza I, Ruiz-Irastorza G. Predictors of peripheral arterial disease in SLE change with patient's age. Lupus Science and Medicine. 2017;4(1) (no pagination)
24) Gao L, Zhao W, Liu Q, Qin M. Association between metabolic syndrome and peripheral arterial disease in elderly patients with type 2 diabetes. Diabetes Metab Syndr Obes. 2021;14:4783-4789
25) Hayfron-Benjamin CF, Maitland-van der Zee AH, van den Born BJ, Amoah AGB, Meeks KAC, Klipstein-Grobusch K, et al. Association between C reactive protein and microvascular and macrovascular dysfunction in sub-Saharan Africans with and without diabetes: the RODAM study. BMJ Open Diabetes Res Care. 2020;8
26) Hayfron-Benjamin CF, Mosterd C, Maitland - van der Zee AH, van Raalte DH, Amoah AGB, Agyemang C, et al. Inflammation and its associations with aortic stiffness, coronary artery disease and peripheral artery disease in different ethnic groups: The HELIUS Study. EClinicalMedicine. 2021;38 (no pagination)
27) Hayfron-Benjamin CF, van den Born B-J, Amoah AGB, Maitland-van der Zee AH, Meeks KAC, Beune EJAJ, et al. Associations of serum uric acid levels with macrovascular and renal microvascular dysfunction among individuals from Sub-Saharan Africa. JAMA Network Open. 2021;4:e2128985-e2128985
28) Hiatt WR, Hoag S, Hamman RF. Effect of diagnostic criteria on the prevalence of peripheral arterial disease. The San Luis Valley Diabetes Study. Circulation. 1995;91:1472-1479
29) Housley E, Leng GC, Donnan PT, Fowkes FG. Physical activity and risk of peripheral arterial disease in the general population: Edinburgh Artery Study. J Epidemiol Community Health. 1993;47:475-480

30) Ix JH, Katz R, De Boer IH, Kestenbaum BR, Allison MA, Siscovick DS, et al. Association of chronic kidney disease with the spectrum of ankle brachial index the CHS (Cardiovascular Health Study). J Am Coll Cardiol. 2009;54:1176-1184
31) Jensen SA, Vatten LJ, Myhre HO. The association between diabetes mellitus and the prevalence of intermittent claudication: the HUNT study. Vasc Med. 2008;13:239-244
32) Jensen SA, Vatten LJ, Nilsen TI, Romundstad PR, Myhre HO. Serum lipids and anthropometric factors related to the prevalence of intermittent claudication. Eur J Vasc Endovasc Surg. 2005;30:582-587
33) Joshy G, Korda RJ, Attia J, Liu B, Bauman AE, Banks E. Body mass index and incident hospitalisation for cardiovascular disease in 158 546 participants from the 45 and Up Study. Int J Obes (Lond). 2014;38:848-856
34) Jouni H, Rodeheffer RJ, Kullo IJ. Increased serum N-terminal pro-B-type natriuretic peptide levels in patients with medial arterial calcification and poorly compressible leg arteries. Arterioscler Thromb Vasc Biol. 2011;31:197-202
35) Kannel WB. Influence of fibrinogen on cardiovascular disease. Drugs. 1997;54 Suppl 3:32-40
36) Knight RJ, Zela S, Schoenberg L, Podder H, Kerman RH, Katz S, et al. The effect of pancreas transplantation on peripheral vascular disease complications. Transplant Proc. 2004;36:1069-1071
37) Ku EJ, Won GW, Lee YH, Lee DH, Jeon HJ, Oh TK. Genetic variation in TCF7L2 rs7903146 correlating with peripheral arterial disease in long-standing type 2 diabetes. Diab Vasc Dis Res. 2020;17:1479164119888475
38) Kullo IJ, Bailey KR, Kardia SL, Mosley TH, Jr., Boerwinkle E, Turner ST. Ethnic differences in peripheral arterial disease in the NHLBI Genetic Epidemiology Network of Arteriopathy (GENOA) study. Vasc Med. 2003;8:237-242
39) Kullo IJ, Shameer K, Jouni H, Lesnick TG, Pathak J, Chute CG, et al. The ATXN2-SH2B3 locus is associated with peripheral arterial disease: an electronic medical record-based genome-wide association study. Front Genet. 2014;5:166
40) Ladehesa-Pineda ML, Arias de la Rosa I, Lopez Medina C, Castro-Villegas MDC, Abalos-Aguilera MDC, Ortega-Castro R, et al. Assessment of the relationship between estimated cardiovascular risk and structural damage in patients with axial spondyloarthritis. Ther Adv Musculoskelet Dis. 2020;12
41) Lee AJ, Fowkes FG, Rattray A, Rumley A, Lowe GD. Haemostatic and rheological factors in intermittent claudication: the influence of smoking and extent of arterial disease. Br J Haematol. 1996;92:226-230
42) Li LX, Lu JX, Shuai HP, Xia HF, Zhang R, Wang JW, et al. Decreased urine uric acid excretion is associated with diabetic retinopathy but not with lower limb atherosclerosis in hospitalized patients with type 2 diabetes. Atherosclerosis. 2015;242:13-18
43) Lin J, Chen Y, Jiang N, Li Z, Xu S. Burden of peripheral artery disease and its attributable risk factors in 204 countries and territories from 1990 to 2019. Front Cardiovasc Med. 2022;9:868370

44) Loprinzi PD, Abbott K. Association of diabetic peripheral arterial disease and objectively-measured physical activity: NHANES 2003-2004. J Diabetes Metab Disord. 2014;13:63
45) Macaulay AC, Montour LT, Adelson N. Prevalence of diabetic and atherosclerotic complications among Mohawk Indians of Kahnawake, PQ. Cmaj. 1988;139:221-224
46) Maggio M, Cattabiani C, Lauretani F, Artoni A, Bandinelli S, Schiavi G, et al. The relationship between sex hormones, sex hormone binding globulin and peripheral artery disease in older persons. Atherosclerosis. 2012;225:469-474
47) Mancini M, Di Donato O, Saldalamacchia G, Liuzzi R, Rivellese A, Salvatore M. Contrast-enhanced ultrasound evaluation of peripheral microcirculation in diabetic patients: effects of cigarette smoking. Radiol Med. 2013;118:206-214
48) Mao Y, Huang Y, Yu H, Xu P, Yu G, Yu J, et al. Incidence of peripheral arterial disease and its association with pulse pressure: a prospective cohort study. Front Endocrinol (Lausanne). 2017;8:333
49) Martelli E, Zamboni M, Sotgiu G, Saderi L, Federici M, Sangiorgi GM, et al. Sex-Related Differences and Factors Associated with Peri-Procedural and 1 Year Mortality in Chronic Limb-Threatening Ischemia Patients from the CLIMATE Italian Registry. J Pers Med. 2023;13(2).
50) Marzban M, Zafarghandi M, Araghi MF, Karimi A, Ahmadi SH, Movahedi N, et al. Impact of diabetes mellitus on peripheral vascular disease concomitant with coronary artery disease. J Tehran Heart Cent. 2009;4(1):39-43
51) Mazidi M, Mikhailidis DP, Banach M. Higher dietary acid load is associated with higher likelihood of peripheral arterial disease among American adults. J Diabetes Complications. 2018;32:565-569
52) Meijer WT, Cost B, Bernsen RM, Hoes AW. Incidence and management of intermittent claudication in primary care in The Netherlands. Scand J Prim Health Care. 2002;20:33-34
53) Mendelson G, Aronow WS, Ahn C. Prevalence of coronary artery disease, atherothrombotic brain infarction, and peripheral arterial disease: associated risk factors in older Hispanics in an academic hospital-based geriatrics practice. J Am Geriatr Soc. 1998;46:481-483
54) Menotti A, Lanti M, Agabiti-Rosei E, Carratelli L, Cavera G, Dormi A, et al. Riskard 2005. New tools for prediction of cardiovascular disease risk derived from Italian population studies. Nutr Metab Cardiovasc Dis. 2005;15:426-440
55) Migdalis IN, Kourti A, Zachariadis D, Voudouris G, Samartzis M. Peripheral vascular disease in newly diagnosed non-insulin-dependent diabetic. Int Angiol. 1992;11:230-232
56) Mogelvang R, Pedersen SH, Flyvbjerg A, Bjerre M, Iversen AZ, Galatius S, et al. Comparison of osteoprotegerin to traditional atherosclerotic risk factors and high-sensitivity C-reactive protein for diagnosis of atherosclerosis. Am J Cardiol. 2012;109:515-520
57) Nachtmann A, Stang A, Wang YM, Wondzinski E, Thilmann AF. Association of obstructive sleep apnea and stenotic artery disease in ischemic stroke patients. Atherosclerosis. 2003;169:301-307

58) Naqvi AZ, Davis RB, Mukamal KJ. Nutrient intake and peripheral artery disease in adults: key considerations in cross-sectional studies. Clin Nutr. 2014;33:443-447
59) Nelson JC, Jiang XC, Tabas I, Tall A, Shea S. Plasma sphingomyelin and subclinical atherosclerosis: findings from the Multi-Ethnic Study of Atherosclerosis. Am J Epidemiol. 2006;163:903-912
60) Noaparast M, Karimian SF, Mirsharifi SR, Rabbani A, Vaezi F. Relation between homocysteine and peripheral vascular atherosclorosis: a brief report. Tehran University Medical Journal. 2013;71(4):259-264
61) Noordzij MJ, Lefrandt JD, Loeffen EA, Saleem BR, Meerwaldt R, Lutgers HL, et al. Skin autofluorescence is increased in patients with carotid artery stenosis and peripheral artery disease. Int J Cardiovasc Imaging. 2012;28:431-438
62) Onofrei V, Adam CA, Marcu DTM, Leon MM, Cumpăt C, Mitu F, et al. Gender Differences and Amputation Risk in Peripheral Artery Disease-A Single-Center Experience. Diagnostics (Basel). 2023;13(19).
63) Palanca A, Castelblanco E, Perpiñán H, Betriu À, Soldevila B, Valdivielso JM, et al. Prevalence and progression of subclinical atherosclerosis in patients with chronic kidney disease and diabetes. Atherosclerosis. 2018;276:50-57
64) Pande RL, Perlstein TS, Beckman JA, Creager MA. Association of insulin resistance and inflammation with peripheral arterial disease: the National Health and Nutrition Examination Survey, 1999 to 2004. Circulation. 2008;118:33-41
65) Perlstein TS, Pande RL, Beckman JA, Creager MA. Serum total bilirubin level and prevalent lower-extremity peripheral arterial disease: National Health and Nutrition Examination Survey (NHANES) 1999 to 2004. Arterioscler Thromb Vasc Biol. 2008;28:166-172
66) Pollex RL, Mamakeesick M, Zinman B, Harris SB, Hanley AJ, Hegele RA. Methylenetetrahydrofolate reductase polymorphism 677C>T is associated with peripheral arterial disease in type 2 diabetes. Cardiovasc Diabetol. 2005;4:17
67) Popa S, Baroi G, Popa RF, Datcu DM, Aursulesei V. Epidemiological characteristics of peripheral artery disease: retrospective study. Rev Med Chir Soc Med Nat Iasi. 2013;117:95-100
68) Prushik SG, Farber A, Gona P, Shrader P, Pencina MJ, D'Agostino RB, Sr., et al. Parental intermittent claudication as risk factor for claudication in adults. Am J Cardiol. 2012;109:736-741
69) Rivera M, Basagaña X, Aguilera I, Foraster M, Agis D, de Groot E, et al. Association between long-term exposure to traffic-related air pollution and subclinical atherosclerosis: the REGICOR study. Environ Health Perspect. 2013;121:223-230
70) Ruff CT, Bhatt DL, Steg PG, Gersh BJ, Alberts MJ, Hoffman EB, et al. Long-term cardiovascular outcomes in patients with atrial fibrillation and atherothrombosis in the REACH registry. Int J Cardiol. 2014;170:413-418
71) Savji N, Rockman CB, Skolnick AH, Guo Y, Adelman MA, Riles T, et al. Association between advanced age and vascular disease in different arterial territories: a population database of over 3.6 million subjects. J Am Coll Cardiol. 2013;61:1736-1743

72) Schroll M. Smoking habits in the Glostrup population of men and women, born in 1914. Implications for health, evaluated from ten-year mortality, incidence of cardiovascular manifestations and pulmonary function, 1964-1974. Acta Med Scand. 1980;208:245-256
73) Shankar A, Klein BE, Nieto FJ, Klein R. Association between serum uric acid level and peripheral arterial disease. Atherosclerosis. 2008;196:749-755
74) Shankar A, Li J, Nieto FJ, Klein BE, Klein R. Association between C-reactive protein level and peripheral arterial disease among US adults without cardiovascular disease, diabetes, or hypertension. Am Heart J. 2007;154:495-501
75) Shankar A, Teppala S, Sabanayagam C. Bisphenol A and peripheral arterial disease: results from the NHANES. Environ Health Perspect. 2012;120:1297-1300
76) Shankar A, Xiao J, Ducatman A. Perfluorooctanoic acid and cardiovascular disease in US adults. Arch Intern Med. 2012;172:1397-1403
77) Signorelli SS, Fiore V, Mangiafico M, Castrogiovanni D. Arterial plaques in peripheral arteries diagnosed by ultrasound in a cohort of patients with type 2 diabetes mellitus: a single-center surveillance. Angiology. 2015;66:675-679
78) Smith FB, Lowe GD, Fowkes FG, Rumley A, Rumley AG, Donnan PT, et al. Smoking, haemostatic factors and lipid peroxides in a population case control study of peripheral arterial disease. Atherosclerosis. 1993;102:155-162
79) Soltau I, Mudersbach E, Geissen M, Schwedhelm E, Winkler MS, Geffken M, et al. Serum-sphingosine-1-phosphate concentrations are inversely associated with atherosclerotic diseases in humans. PLoS One. 2016;11:e0168302
80) Stein RA, Rockman CB, Guo Y, Adelman MA, Riles T, Hiatt WR, et al. Association between physical activity and peripheral artery disease and carotid artery stenosis in a self-referred population of 3 million adults. Arterioscler Thromb Vasc Biol. 2015;35:206-212
81) Strobl FF, Rominger A, Wolpers S, Rist C, Bamberg F, Thierfelder KM, et al. Impact of cardiovascular risk factors on vessel wall inflammation and calcified plaque burden differs across vascular beds: a PET-CT study. Int J Cardiovasc Imaging. 2013;29:1899-1908
82) Suarez-Zdunek MA, Høgh J, Kirkegaard-Klitbo DM, Jensen AMR, Rupert A, Trøseid M, et al. High incidence of subclinical peripheral artery disease in people with HIV. Aids. 2022
83) Sung JH, Lee JE, Samdarshi TE, Nagarajarao HS, Taylor JK, Agrawal KK, et al. C-reactive protein and subclinical cardiovascular disease among African-Americans: the Jackson Heart Study. J Cardiovasc Med (Hagerstown). 2014;15:371-376
84) Taylor LM, Jr., DeFrang RD, Harris EJ, Jr., Porter JM. The association of elevated plasma homocyst(e)ine with progression of symptomatic peripheral arterial disease. J Vasc Surg. 1991;13:128-136
85) Tian LB, Fang H, Gao L, Tan Z, Zhen YF, Tian JL, et al. 9p21 polymorphisms increase the risk of peripheral artery disease in the Han Chinese population. J Int Med Res. 2013;41:106-114

86) Tovillas FJ, Dalfó A, Romea S, Sisó A, Senar E, Miracle M. Cardiovascular morbidity and its relationship to left ventricular hypertrophy in a cohort of hypertensive patients: the Gòtic study. Aten Primaria. 2001;28:315-319
87) Tseng CH, Chong CK, Chen CJ, Tai TY. Dose-response relationship between peripheral vascular disease and ingested inorganic arsenic among residents in blackfoot disease endemic villages in Taiwan. Atherosclerosis. 1996;120:125-133
88) Tseng CH, Chong CK, Chen CJ, Tai TY. Lipid profile and peripheral vascular disease in arseniasis-hyperendemic villages in Taiwan. Angiology. 1997;48:321-335
89) Tseng CH, Huang YK, Huang YL, Chung CJ, Yang MH, Chen CJ, et al. Arsenic exposure, urinary arsenic speciation, and peripheral vascular disease in blackfoot disease-hyperendemic villages in Taiwan. Toxicol Appl Pharmacol. 2005;206:299-308
90) Tseng CH. Lipoprotein(a) is an independent risk factor for peripheral arterial disease in Chinese type 2 diabetic patients in Taiwan. Diabetes Care. 2004;27:517-521
91) Tseng CH. Pulse pressure as a risk factor for peripheral vascular disease in type 2 diabetic patients. Clin Exp Hypertens. 2003;25:475-485
92) Vaccaro O, Pauciullo P, Rubba P, Annuzzi G, Rivellese AA, Riccardi G, et al. Peripheral arterial circulation in individuals with impaired glucose tolerance. Diabetes Care. 1985;8:594-597
93) Vidula H, Liu K, Criqui MH, Szklo M, Allison M, Sibley C, et al. Metabolic syndrome and incident peripheral artery disease - the Multi-Ethnic Study of Atherosclerosis. Atherosclerosis. 2015;243:198-203
94) Voulgari C, Katsilambros N, Tentolouris N. Smoking cessation predicts amelioration of microalbuminuria in newly diagnosed type 2 diabetes mellitus: a 1-year prospective study. Metabolism. 2011;60:1456-1464
95) Wick PA, Mombelli A, Pagano S, Moren X, Giannopoulou C, Mach F, et al. Anti-apolipoprotein A-1 autoantibodies as biomarker for atherosclerosis burden in patients with periodontitis. J Periodontal Res. 2013;48:350-356
96) Woo J, Yu BWM, Chan RSM, Leung J. Influence of dietary patterns and inflammatory markers on atherosclerosis using ankle brachial index as a surrogate. J Nutr Health Aging. 2018;22:619-626
97) Xie X, Ma YT, Yang YN, Li XM, Liu F, Huang D, et al. Alcohol consumption and ankle-to-brachial index: results from the Cardiovascular Risk Survey. PLoS One. 2010;5:e15181
98) Yang S, Wang S, Yang B, Zheng J, Cai Y, Yang Z. Alcohol consumption is a risk factor for lower extremity arterial disease in Chinese patients with T2DM. J Diabetes Res. 2017;2017:8756978
99) Yao CW, Shen TC, Lu CR, Wang YC, Lin CL, Tu CY, et al. Asthma is associated with a subsequent risk of peripheral artery disease: a longitudinal population-based study. Medicine (Baltimore). 2016;95:e2546
100) Yu JH, Hwang JY, Shin MS, Jung CH, Kim EH, Lee SA, et al. The prevalence of peripheral arterial disease in korean patients with type 2 diabetes mellitus attending a university hospital. Diabetes Metab J. 2011;35:543-550

101) Yuan J, Jia P, Hua L, Xin Z, Yang JK. Vitamin D deficiency is associated with risk of developing peripheral arterial disease in type 2 diabetic patients. BMC Cardiovasc Disord. 2019;19:145
102) Zalawadiya SK, Veeranna V, Panaich SS, Afonso L. Red cell distribution width and risk of peripheral artery disease: analysis of National Health and Nutrition Examination Survey 1999-2004. Vasc Med. 2012;17:155-163
103) Zamzam A, Syed MH, Harlock J, Eikelboom J, Singh KK, Abdin R, et al. Urinary fatty acid binding protein 3 (uFABP3) is a potential biomarker for peripheral arterial disease. Sci Rep. 2021;11:11061
104) Zhan Y, Dong Y, Tang Z, Zhang F, Hu D, Yu J. Serum uric acid, gender, and low ankle brachial index in adults with high cardiovascular risk. Angiology. 2015;66:687-691
105) Zhang Y, Guo Y, Shen X, Zhao F, Yan S. Lower body mass index is not of more benefit for diabetic complications. J Diabetes Investig. 2019;10:1307-1317
106) Zhang Y, Peng K, Liu J, Chen X, Wang T, Li M, et al. Carotid intima-media thickness and plagues are associated with indicators of peripheral artery diseases in patients with diabetes. Diabetes Res Clin Pract. 2018;144:245-251
107) Zou SY, Zhao Y, Shen YP, Shi YF, Zhou HJ, Zou JY, et al. Identifying at-risk foot among hospitalized patients with type 2 diabetes: a cross-sectional study in one Chinese tertiary hospital. Chronic Dis Transl Med. 2015;1(4):210-216

Review or editorial articles (n=3)

1) Belch JJ. Metabolic, endocrine and haemodynamic risk factors in the patient with peripheral arterial disease. Diabetes Obes Metab. 2002;4 Suppl 2:S7-13
2) Miedema MD. Assessment of subclinical atherosclerosis in smokers: does lower risk mean low risk? JACC Cardiovasc Imaging. 2019;12:1818-1820
3) Thierer J. Differences in cardiovascular health in men and women. An analysis of the PURE registry. Revista Argentina de Cardiologia. 2020;88(4):390-399

Smoking as a risk factor for peripheral arterial disease in univariable or multivariable analyses, not by sex (n=164)

1) Agarwal AK, Singh M, Arya V, Garg U, Singh VP, Jain V. Prevalence of peripheral arterial disease in type 2 diabetes mellitus and its correlation with coronary artery disease and its risk factors. J Assoc Physicians India. 2012;60:28-32
2) Agner E. Smoking and health in old age. A ten-year follow-up study. Acta Med Scand. 1985;218:311-316
3) Akram J, Aamir AU, Basit A, Qureshi MS, Mehmood T, Shahid SK, et al. Prevalence of peripheral arterial disease in type 2 diabetics in Pakistan. J Pak Med Assoc. 2011;61:644-648
4) Alcolado JC, Pacy PJ, Beevers M, Dodson PM. Risk factors for peripheral vascular disease in hypertensive subjects with type 2 diabetes mellitus. Diabet Med. 1992;9:904-907

5) Al-Khawlani A, Atef ZA, Al-Ansi A. Macrovascular complications and their associated risk factors in type 2 diabetic patients in Sana'a city, Yemen. East Mediterr Health J. 2010;16:851-858
6) Allison MA, Hiatt WR, Hirsch AT, Coll JR, Criqui MH. A high ankle-brachial index is associated with increased cardiovascular disease morbidity and lower quality of life. J Am Coll Cardiol. 2008;51:1292-1298
7) Al-Sheikh SO, Aljabri BA, Al-Ansary LA, Al-Khayal LA, Al-Salman MM, Al-Omran MA. Prevalence of and risk factors for peripheral arterial disease in Saudi Arabia. A pilot cross-sectional study. Saudi Med J. 2007;28:412-414
8) Amidou SA, Houehanou YC, Houinato SD, Aboyans V, Sonou A, Saka D, et al. Epidemiology of lower extremity artery disease in a rural setting in Benin, West Africa: the TAHES study. Int J Cardiol. 2018;267:198-201
9) Anjani Kumar PVL, Sindhura P, Chaitanya S, Prabhakar D, Narender B, Bakshi V. Determination of peripheral artery disease using ankle-brachial index. Asian Journal of Pharmaceutics. 2018;12(4 Supplement):S1345-S1355
10) Antonopoulos S, Kokkoris S, Stasini F, Mylonopoulou M, Lepeniotis G, Mikros S, et al. High prevalence of subclinical peripheral artery disease in Greek hospitalized patients. Eur J Intern Med. 2005;16:187-191
11) Aponte J. The prevalence of asymptomatic and symptomatic peripheral arterial disease and peripheral arterial disease risk factors in the U.S. population. Holist Nurs Pract. 2011;25:147-161
12) Azhar A, Basheer M, Abdelgawad MS, Roshdi H, Kamel MF. Prevalence of peripheral arterial disease in diabetic foot ulcer patients and its impact in limb salvage. Int J Low Extrem Wounds. 2021:15347346211027063
13) Bahadori B, Uitz E, Dehchamani D, Pilger E, Renner W. The fibrinogen gamma 10034C>T polymorphism is not associated with peripheral arterial disease. Thromb Res. 2010;126:350-352
14) Bahadori B, Uitz E, Mayer A, Harauer J, Dam K, Truschnig-Wilders M, et al. Polymorphisms of the hypoxia-inducible factor 1 gene and peripheral artery disease. Vasc Med. 2010;15:371-374
15) Beach KW, Bedford GR, Bergelin RO, Martin DC, Vandenberghe N, Zaccardi M, et al. Progression of lower-extremity arterial occlusive disease in type II diabetes mellitus. Diabetes Care. 1988;11:464-472
16) Bennett PC, Lip GY, Silverman S, Blann AD, Gill PS. The contribution of cardiovascular risk factors to peripheral arterial disease in South Asians and Blacks: a sub-study to the Ethnic-Echocardiographic Heart of England Screening (E-ECHOES) study. Qjm. 2010;103:661-669
17) Bérard AM, Bedel A, Le Trequesser R, Freyburger G, Nurden A, Colomer S, et al. Novel risk factors for premature peripheral arterial occlusive disease in non-diabetic patients: a case-control study. PLoS One. 2013;8:e37882
18) Berger JS, Hochman J, Lobach I, Adelman MA, Riles TS, Rockman CB. Modifiable risk factor burden and the prevalence of peripheral artery disease in different vascular territories. J Vasc Surg. 2013;58:673-681.e671

19) Bertoia ML, Pai JK, Lee JH, Taleb A, Joosten MM, Mittleman MA, et al. Oxidation-specific biomarkers and risk of peripheral artery disease. J Am Coll Cardiol. 2013;61:2169-2179
20) Bertoni AG, Whitt-Glover MC, Chung H, Le KY, Barr RG, Mahesh M, et al. The association between physical activity and subclinical atherosclerosis: the Multi-Ethnic Study of Atherosclerosis. Am J Epidemiol. 2009;169:444-454
21) Bianchi C, Penno G, Pancani F, Civitelli A, Piaggesi A, Caricato F, et al. Non-traditional cardiovascular risk factors contribute to peripheral arterial disease in patients with type 2 diabetes. Diabetes Res Clin Pract. 2007;78:246-253
22) Bradby GV, Valente AJ, Walton KW. Serum high-density lipoproteins in peripheral vascular disease. Lancet. 1978;2:1271-1274
23) Brevetti G, Oliva G, Silvestro A, Scopacasa F, Chiariello M. Prevalence, risk factors and cardiovascular comorbidity of symptomatic peripheral arterial disease in Italy. Atherosclerosis. 2004;175:131-138
24) Carbayo JA, Divisón JA, Escribano J, López-Abril J, López de Coca E, Artigao LM, et al. Using ankle-brachial index to detect peripheral arterial disease: prevalence and associated risk factors in a random population sample. Nutr Metab Cardiovasc Dis. 2007;17:41-49
25) Carvounis CP, Nikas N. Prevalence of peripheral arterial disease in subjects at moderate cardiovascular risk: Greek results of the PANDORA study. Hellenic J Cardiol. 2014;55:294-304
26) Chen J, Mohler ER, 3rd, Xie D, Shlipak MG, Townsend RR, Appel LJ, et al. Risk factors for peripheral arterial disease among patients with chronic kidney disease. Am J Cardiol. 2012;110:136-141
27) Chen J, Mohler ER, Xie D, Shlipak M, Townsend RR, Appel LJ, et al. Traditional and non-traditional risk factors for incident peripheral arterial disease among patients with chronic kidney disease. Nephrol Dial Transplant. 2016;31:1145-1151
28) Cheng SW, Ting AC, Wong J. Lipoprotein (a) and its relationship to risk factors and severity of atherosclerotic peripheral vascular disease. Eur J Vasc Endovasc Surg. 1997;14:17-23
29) Chuengsamarn S, Sangpanich A, Laoopugsin N. Prevalence and risk factors of peripheral arterial disease in type 2 diabetic patients at HRH Princess Maha Chakri Sirindhorn Medical Center. J Med Assoc Thai. 2010;93 Suppl 2:S32-38
30) Coni N, Tennison B, Troup M. Prevalence of lower extremity arterial disease among elderly people in the community. Br J Gen Pract. 1992;42:149-152
31) Cornejo Del Río V, Mostaza J, Lahoz C, Sánchez-Arroyo V, Sabín C, López S, et al. Prevalence of peripheral artery disease (PAD) and factors associated: an epidemiological analysis from the population-based Screening PRE-diabetes and type 2 DIAbetes (SPREDIA-2) study. PLoS One. 2017;12:e0186220
32) da Silva Filho PJ, Martinez Teodoro EC, Alves Pereira EC, dos Reis Miranda VC. Prevalence of peripheral arterial disease and associated factors in people with type 2 diabetes. Fisioterapia em Movimento. 2021;34:1-12

33) de Vinuesa SG, Ortega M, Martinez P, Goicoechea M, Campdera FG, Luño J. Subclinical peripheral arterial disease in patients with chronic kidney disease: prevalence and related risk factors. Kidney Int Suppl. 2005:S44-47
34) Desormais I, Aboyans V, Guerchet M, Ndamba-Bandzouzi B, Mbelesso P, Dantoine T, et al. Prevalence of peripheral artery disease in the elderly population in urban and rural areas of Central Africa: the EPIDEMCA study. Eur J Prev Cardiol. 2015;22:1462-1472
35) Dieter RS, Tomasson J, Gudjonsson T, Brown RL, Vitcenda M, Einerson J, et al. Lower extremity peripheral arterial disease in hospitalized patients with coronary artery disease. Vasc Med. 2003;8:233-236
36) Donnan PT, Thomson M, Fowkes FG, Prescott RJ, Housley E. Diet as a risk factor for peripheral arterial disease in the general population: the Edinburgh Artery Study. Am J Clin Nutr. 1993;57:917-921
37) Eraso LH, Fukaya E, Mohler ER, 3rd, Xie D, Sha D, Berger JS. Peripheral arterial disease, prevalence and cumulative risk factor profile analysis. Eur J Prev Cardiol. 2014;21:704-711
38) Escobar C, Blanes I, Ruiz A, Vinuesa D, Montero M, Rodríguez M, et al. Prevalence and clinical profile and management of peripheral arterial disease in elderly patients with diabetes. Eur J Intern Med. 2011;22:275-281
39) Fabsitz RR, Sidawy AN, Go O, Lee ET, Welty TK, Devereux RB, et al. Prevalence of peripheral arterial disease and associated risk factors in American Indians: the Strong Heart Study. Am J Epidemiol. 1999;149:330-338
40) Fatemi S, Gottsäter A, Zarrouk M, Engström G, Melander O, Persson M, et al. Lp-PLA(2) activity and mass and CRP are associated with incident symptomatic peripheral arterial disease. Sci Rep. 2019;9:5609
41) Félix-Redondo FJ, Fernández-Bergés D, Grau M, Baena-Diez JM, Mostaza JM, Vila J. Prevalence and clinical characteristics of peripheral arterial disease in the study population Hermex. Rev Esp Cardiol (Engl Ed). 2012;65:726-733
42) Fowkes FG, Connor JM, Smith FB, Wood J, Donnan PT, Lowe GD. Fibrinogen genotype and risk of peripheral atherosclerosis. Lancet. 1992;339:693-696
43) Fowkes FG, Housley E, Riemersma RA, Macintyre CC, Cawood EH, Prescott RJ, et al. Smoking, lipids, glucose intolerance, and blood pressure as risk factors for peripheral atherosclerosis compared with ischemic heart disease in the Edinburgh Artery Study. Am J Epidemiol. 1992;135:331-340
44) Fowkes FG, Thorogood M, Connor MD, Lewando-Hundt G, Tzoulaki I, Tollman SM. Distribution of a subclinical marker of cardiovascular risk, the ankle brachial index, in a rural African population: SASPI study. Eur J Cardiovasc Prev Rehabil. 2006;13:964-969
45) Franck M, Staub HL, Petracco JB, Norman GL, Lassen AJ, Schiavo N, et al. Autoantibodies to the atheroma component beta2-glycoprotein I and risk of symptomatic peripheral artery disease. Angiology. 2007;58:295-302
46) Garofolo L, Ferreira SR, Miranda Junior F. Study of risk factors associated with peripheral arteriopathy in Japanese-Brazilians from Bauru (SP). Arq Bras Cardiol. 2014;102:143-150

47) Gofin R, Kark JD, Friedlander Y, Lewis BS, Witt H, Stein Y, et al. Peripheral vascular disease in a middle-aged population sample. The Jerusalem Lipid Research Clinic Prevalence Study. Isr J Med Sci. 1987;23:157-167
48) Guan H, Li YJ, Xu ZR, Li GW, Guo XH, Liu ZM, et al. Prevalence and risk factors of peripheral arterial disease in diabetic patients over 50 years old in China. Chin Med Sci J. 2007;22:83-88
49) Hooi JD, Kester AD, Stoffers HE, Overdijk MM, van Ree JW, Knottnerus JA. Incidence of and risk factors for asymptomatic peripheral arterial occlusive disease: a longitudinal study. Am J Epidemiol. 2001;153:666-672
50) Hooi JD, Stoffers HE, Kester AD, Rinkens PE, Kaiser V, van Ree JW, et al. Risk factors and cardiovascular diseases associated with asymptomatic peripheral arterial occlusive disease. The Limburg PAOD Study. Peripheral Arterial Occlusive Disease. Scand J Prim Health Care. 1998;16:177-182
51) Howard DP, Banerjee A, Fairhead JF, Hands L, Silver LE, Rothwell PM. Population-based study of incidence, risk factors, outcome, and prognosis of ischemic peripheral arterial events: implications for prevention. Circulation. 2015;132:1805-1815
52) Huang KC, Chen CC, Su YC, Lin JS, Chang CT, Wang TY, et al. The relationship between stasis-stagnation constitution and peripheral arterial disease in patients with type 2 diabetes. Evid Based Complement Alternat Med. 2014;2014:903798
53) Hughson WG, Mann JI, Garrod A. Intermittent claudication: prevalence and risk factors. Br Med J. 1978;1:1379-1381
54) Huh JH, Choi E, Lim JS, Lee MY, Chung CH, Shin JY. Serum cystatin C levels are associated with asymptomatic peripheral arterial disease in type 2 diabetes mellitus patients without overt nephropathy. Diabetes Res Clin Pract. 2015;108:258-264
55) Ishimura E, Okuno S, Kitatani K, Kim M, Shoji T, Nakatani T, et al. Different risk factors for peripheral vascular calcification between diabetic and non-diabetic haemodialysis patients--importance of glycaemic control. Diabetologia. 2002;45:1446-1448
56) Jackson SL, Safo S, Staimez LR, Long Q, Rhee MK, Cunningham SA, et al. Reduced cardiovascular disease incidence with a national lifestyle change program. Am J Prev Med. 2017;52:459-468
57) Kapoor R, Ayers C, Visotcky A, Mason P, Kulinski J. Association of sex and height with a lower ankle brachial index in the general population. Vasc Med. 2018;23:534-540
58) Kennedy M, Solomon C, Manolio TA, Criqui MH, Newman AB, Polak JF, et al. Risk factors for declining ankle-brachial index in men and women 65 years or older: the Cardiovascular Health Study. Arch Intern Med. 2005;165:1896-1902
59) Khaleghi M, Kullo IJ. Aortic augmentation index is associated with the ankle-brachial index: a community-based study. Atherosclerosis. 2007;195:248-253
60) Konin C, Essam N'loo A S, Adoubi A, Coulibaly I, N'Guetta R, Boka B, et al. Peripheral arterial disease of the lower limbs in African diabetic patients: ultrasonography and determining factors. J Mal Vasc. 2014;39:373-381
61) Korhonen P, Kautiainen H, Aarnio P. Pulse pressure and subclinical peripheral artery disease. J Hum Hypertens. 2014;28:242-245

62) Kravos A, Bubnic-Sotosek K. Ankle-brachial index screening for peripheral artery disease in asymptomatic patients between 50 and 70 years of age. J Int Med Res. 2009;37:1611-1619
63) Krishnan MN, Geevar Z, Mohanan PP, Venugopal K, Devika S. Prevalence of peripheral artery disease and risk factors in the elderly: a community based cross-sectional study from northern Kerala, India. Indian Heart J. 2018;70:808-815
64) Kröger K, Dragano N, Stang A, Moebus S, Möhlenkamp S, Mann K, et al. An unequal social distribution of peripheral arterial disease and the possible explanations: results from a population-based study. Vasc Med. 2009;14:289-296
65) Kröger K, Lehmann N, Moebus S, Schmermund A, Stang A, Kälsch H, et al. Impact of atherosclerotic risk factors on different ankle-brachial-index criteria--results of the Heinz Nixdorf RECALL study. Vasa. 2013;42:120-126
66) Kumar A, Al-Bader M, Al-Thani H, El-Menyar A, Al Suwaidi J, Al-Zakwani I, et al. Multicenter cross-sectional study of asymptomatic peripheral arterial disease among patients with a single previous coronary or cerebrovascular event in the Arabian Gulf. Curr Med Res Opin. 2014;30:1725-1732
67) Kumar A, Mash B, Rupesinghe G. Peripheral arterial disease - high prevalence in rural black South Africans. S Afr Med J. 2007;97:285-288
68) Lane JS, Magno CP, Lane KT, Chan T, Hoyt DB, Greenfield S. Nutrition impacts the prevalence of peripheral arterial disease in the United States. J Vasc Surg. 2008;48:897-904
69) Lapice E, Cipriano P, Patti L, Romano G, Vaccaro O, Rivellese AA. Fasting apolipoprotein B48 is associated with asymptomatic peripheral arterial disease in type 2 diabetic subjects: a case-control study. Atherosclerosis. 2012;223:504-506
70) Lema-Verdia L, Balboa-Barreiroa V, Couceiro-Sanchez E, Gonzalez-Martin C, Pertega-Diaz S, Seoane-Pillado T, et al. Peripheral arterial disease in renal transplant patients. Validity of the edinburgh questionnaire for disease diagnosis. Revista de Nefrologia, Dialisis y Trasplante. 2021;41(2):100-112
71) Leng GC, Horrobin DF, Fowkes FG, Smith FB, Lowe GD, Donnan PT, et al. Plasma essential fatty acids, cigarette smoking, and dietary antioxidants in peripheral arterial disease. A population-based case-control study. Arterioscler Thromb. 1994;14:471-478
72) Leng GC, Lee AJ, Fowkes FG, Lowe GD, Housley E. The relationship between cigarette smoking and cardiovascular risk factors in peripheral arterial disease compared with ischaemic heart disease. The Edinburgh Artery Study. Eur Heart J. 1995;16:1542-1548
73) Leng GC, Papacosta O, Whincup P, Wannamethee G, Walker M, Ebrahim S, et al. Femoral atherosclerosis in an older British population: prevalence and risk factors. Atherosclerosis. 2000;152:167-174
74) Leong BD, Ariffin AZ, Chuah JA, Voo SY. Prevalence of peripheral arterial disease and abdominal aortic aneurysm among patients with acute coronary syndrome. Med J Malaysia. 2013;68:10-12
75) Li J, Luo Y, Xu Y, Yang J, Zheng L, Hasimu B, et al. Risk factors of peripheral arterial disease and relationship between low ankle - brachial index and mortality from all-

cause and cardiovascular disease in Chinese patients with type 2 diabetes. Circ J. 2007;71:377-381
76) Li R, Nicklas B, Pahor M, Newman A, Sutton-Tyrrell K, Harris T, et al. Polymorphisms of angiotensinogen and angiotensin-converting enzyme associated with lower extremity arterial disease in the Health, Aging and Body Composition study. J Hum Hypertens. 2007;21:673-682
77) Liang KP, Liang KV, Matteson EL, McClelland RL, Christianson TJ, Turesson C. Incidence of noncardiac vascular disease in rheumatoid arthritis and relationship to extraarticular disease manifestations. Arthritis Rheum. 2006;54:642-648
78) Lilja E, Bergwall S, Sonestedt E, Gottsäter A, Acosta S. The association between dietary intake, lifestyle and incident symptomatic peripheral arterial disease among individuals with diabetes mellitus: insights from the Malmö Diet and Cancer study. Ther Adv Endocrinol Metab. 2019;10:2042018819890532
79) Liu MC, Lee YW, Lee PT, Chang CS, Tai YL, Yu JR, et al. Cyclophilin A is associated with peripheral artery disease and chronic kidney disease in geriatrics: The Tianliao Old People (TOP) study. Sci Rep. 2015;5:9937
80) Luo YY, Li J, Xin Y, Zheng LQ, Yu JM, Hu DY. Risk factors of peripheral arterial disease and relationship between low ankle brachial index and mortality from all-cause and cardiovascular disease in Chinese patients with hypertension. J Hum Hypertens. 2007;21:461-466
81) MacGregor AS, Price JF, Hau CM, Lee AJ, Carson MN, Fowkes FG. Role of systolic blood pressure and plasma triglycerides in diabetic peripheral arterial disease. The Edinburgh Artery Study. Diabetes Care. 1999;22:453-458
82) Mary A, Hartemann A, Liabeuf S, Aubert CE, Kemel S, Salem JE, et al. Association between metformin use and below-the-knee arterial calcification score in type 2 diabetic patients. Cardiovasc Diabetol. 2017;16:24
83) Masse M, Hébert MJ, Troyanov S, Vigneault N, Sirois I, Madore F. Soluble Fas is a marker of peripheral arterial occlusive disease in haemodialysis patients. Nephrol Dial Transplant. 2002;17:485-491
84) Matsushita K, Sang Y, Ning H, Ballew SH, Chow EK, Grams ME, et al. Lifetime risk of lower-extremity peripheral artery disease defined by ankle-brachial index in the United States. J Am Heart Assoc. 2019;8:e012177
85) Mattei J, Sotres-Alvarez D, Gellman M, Castañeda SF, Hu FB, Tucker KL, et al. Diet quality, inflammation, and the ankle brachial index in adults with or without cardiometabolic conditions. Clin Nutr. 2018;37:1332-1339
86) Mazoyer E, Drouet L, Soria C, Fruchard JC, Pellerin A, Arcan JC, et al. Risk factors and outcomes for atherothrombotic disease in French patients: the RIVAGE study. RIsque VAsculaire Group d'Etude. Thromb Res. 1999;95:163-176
87) McDermott MM, Guralnik JM, Corsi A, Albay M, Macchi C, Bandinelli S, et al. Patterns of inflammation associated with peripheral arterial disease: the InCHIANTI study. Am Heart J. 2005;150:276-281
88) McDermott MM, Kerwin DR, Liu K, Martin GJ, O'Brien E, Kaplan H, et al. Prevalence and significance of unrecognized lower extremity peripheral arterial disease in general medicine practice. J Gen Intern Med. 2001;16:384-390

89) Meijer WT, Grobbee DE, Hunink MG, Hofman A, Hoes AW. Determinants of peripheral arterial disease in the elderly: the Rotterdam study. Arch Intern Med. 2000;160:2934-2938
90) Mejias SG, Ramphul K. Prevalence of peripheral arterial disease among diabetic patients in Santo Domingo, Dominican Republic and associated risk factors. Arch Med Sci Atheroscler Dis. 2018;3:e35-e40
91) Milne JS, Williamson J. Intermittent claudication and peripheral pulses in older people. Age Ageing. 1972;1:146-151
92) Murabito JM, D'Agostino RB, Silbershatz H, Wilson WF. Intermittent claudication. A risk profile from The Framingham Heart Study. Circulation. 1997;96:44-49
93) Murabito JM, Evans JC, Nieto K, Larson MG, Levy D, Wilson PW. Prevalence and clinical correlates of peripheral arterial disease in the Framingham Offspring Study. Am Heart J. 2002;143:961-965
94) Nag F, De A, Hazra A, Chatterjee G, Ghosh A, Surana TV. Chronic venous ulceration of leg associated with peripheral arterial disease: an underappreciated entity in developing country. Int Wound J. 2014;11:546-549
95) Neamah HR, Hassan QA, Kamber HM. Prevalence of peripheral arterial disease in end stage renal disease patients undergoing hemodialysis: a cross-sectional study. Pakistan Journal of Medical and Health Sciences. 2018;12(1):474-477
96) Newman AB, Sutton-Tyrrell K, Kuller LH. Lower-extremity arterial disease in older hypertensive adults. Arterioscler Thromb. 1993;13:555-562
97) Newman AB, Sutton-Tyrrell K, Rutan GH, Locher J, Kuller LH. Lower extremity arterial disease in elderly subjects with systolic hypertension. J Clin Epidemiol. 1991;44:15-20
98) Newman JD, Navas-Acien A, Kuo CC, Guallar E, Howard BV, Fabsitz RR, et al. Peripheral arterial disease and its association with arsenic exposure and metabolism in the Strong Heart Study. Am J Epidemiol. 2016;184:806-817
99) Niu Y, Zhang W, Yang Z, Li X, Wen J, Wang S, et al. Association of plasma osteoprotegerin levels with the severity of lower extremity arterial disease in patients with type 2 diabetes. BMC Cardiovasc Disord. 2015;15:86
100) Ohori K, Yano T, Katano S, Kouzu H, Inoue T, Takamura Y, et al. Independent link between peripheral artery disease and muscle wasting in patients with heart failure. ESC Heart Fail. 2020;7:3252-3256
101) Ostchega Y, Paulose-Ram R, Dillon CF, Gu Q, Hughes JP. Prevalence of peripheral arterial disease and risk factors in persons aged 60 and older: data from the National Health and Nutrition Examination Survey 1999-2004. J Am Geriatr Soc. 2007;55:583-589
102) Ozeki M, Morita H, Miyamura M, Fujisaka T, Fujita SI, Ito T, et al. High serum bilirubin is associated with lower prevalence of peripheral arterial disease among cardiac patients. Clin Chim Acta. 2018;476:60-66
103) Paisey RB, Arredondo G, Villalobos A, Lozano O, Guevara L, Kelly S. Association of differing dietary, metabolic, and clinical risk factors with macrovascular complications of diabetes: a prevalence study of 503 Mexican type II diabetic subjects. I. Diabetes Care. 1984;7:421-427

104) Panico MDB, Spichler ES, Neves MF, Pinto LW, Spichler D. Prevalence and risk factors of symptomatic and asymptomatic peripheral arterial disease in a tertiary care hospital, Rio de Janeiro, Brazil. J Vasc Bras. 2009;8(2):125-132
105) Passos VM, Barreto SM, Guerra HL, Firmo JO, Vidigal PG, Lima-Costa MF. The Bambuí health and aging study (BHAS). Prevalence of intermittent claudication in the aged population of the community of Bambuí and its associated factors. Arq Bras Cardiol. 2001;77:453-462
106) Pieper B, Kirsner RS, Templin TN, Birk TJ. Peripheral arterial disease among substance abusers in drug treatment. Adv Skin Wound Care. 2009;22:265-272
107) Powell JT, Edwards RJ, Worrell PC, Franks PJ, Greenhalgh RM, Poulter NR. Risk factors associated with the development of peripheral arterial disease in smokers: a case-control study. Atherosclerosis. 1997;129:41-48
108) Price JF, Lee AJ, Fowkes FG. Hyperinsulinaemia: a risk factor for peripheral arterial disease in the non-diabetic general population. J Cardiovasc Risk. 1996;3:501-505
109) Price JF, Mowbray PI, Lee AJ, Rumley A, Lowe GD, Fowkes FG. Relationship between smoking and cardiovascular risk factors in the development of peripheral arterial disease and coronary artery disease: Edinburgh Artery Study. Eur Heart J. 1999;20:344-353
110) Rabia K, Khoo EM. Prevalence of peripheral arterial disease in patients with diabetes mellitus in a primary care setting. Med J Malaysia. 2007;62:130-133
111) Rajagopalan S, Dellegrottaglie S, Furniss AL, Gillespie BW, Satayathum S, Lameire N, et al. Peripheral arterial disease in patients with end-stage renal disease: observations from the Dialysis Outcomes and Practice Patterns Study (DOPPS). Circulation. 2006;114:1914-1922
112) Reiner Ž, De Sutter J, Ryden L, Mirrakhimov E, Pogosova N, Dolzhenko M, et al. Peripheral arterial disease and intermittent claudication in coronary heart disease patients. Int J Cardiol. 2021;322:227-232
113) Rerkasem A, Sripan P, Pongtam S, Ounjaijean S, Kulprachakarn K, Wongthanee A, et al. The prevalence and risk factors for peripheral arterial disease in adults living with human immunodeficiency virus. Int J Low Extrem Wounds. 2021:15347346211009404
114) Rong D, Liu J, Jia X, Al-Nafisee D, Jia S, Sun G, et al. Hyperhomocysteinaemia is an independent risk factor for peripheral arterial disease in a Chinese Han population. Atherosclerosis. 2017;263:205-210
115) Sabino AP, De Oliveira Sousa M, Moreira Lima L, Dias Ribeiro D, Sant'Ana Dusse LM, Das Graças Carvalho M, et al. ApoB/ApoA-I ratio in young patients with ischemic cerebral stroke or peripheral arterial disease. Transl Res. 2008;152:113-118
116) Sadrzadeh Rafie AH, Stefanick ML, Sims ST, Phan T, Higgins M, Gabriel A, et al. Sex differences in the prevalence of peripheral artery disease in patients undergoing coronary catheterization. Vasc Med. 2010;15:443-450
117) Saleh A, Makhamreh H, Qoussoos T, Alawwa I, Alsmady M, Salah ZA, et al. Prevalence of previously unrecognized peripheral arterial disease in patients undergoing coronary angiography. Medicine (Baltimore). 2018;97:e11519

118) Samman Tahhan A, Hayek SS, Sandesara P, Hajjari J, Hammadah M, O'Neal WT, et al. Circulating soluble urokinase plasminogen activator receptor levels and peripheral arterial disease outcomes. Atherosclerosis. 2017;264:108-114
119) Sari R, Balci MK. Relationship between C peptide and chronic complications in type-2 diabetes mellitus. J Natl Med Assoc. 2005;97:1113-1118
120) Seki J, Ohashi M, Sato T, Yamamoto M, Fujii S, Wada M. Peripheral vascular disease in Japanese diabetics: screening by the Doppler ultrasonic technique. Tohoku J Exp Med. 1983;141 Suppl:499-506
121) Selvin E, Erlinger TP. Prevalence of and risk factors for peripheral arterial disease in the United States: results from the National Health and Nutrition Examination Survey, 1999-2000. Circulation. 2004;110:738-743
122) Serve E, Reny JL, Akhavan S, Emmerich J, Fischer AM, Tapon-Bretaudière J. A two adenine insertion polymorphism in the 3' untranslated region of factor VII gene is associated with peripheral arterial disease but not with venous thrombosis. Results of case-control studies. Thromb Haemost. 2007;98:733-737
123) Sheng CS, Huang QF, Huang JF, Li Y, Wang JG. Prevalence and related factors of abnormal four-limb blood pressure difference in elderly people in Shanghai. Journal of Shanghai Jiaotong University (Medical Science). 2019;39(3):287-291
124) Shi M, Jiang W, Wang Z, Shi W, Sun R, Chen E, et al. Ultrasonic examination of carotid artery and peripheral artery atherosclerosis and their risk factors. Journal of Jilin University Medicine Edition. 2015;41(5):1055-1059
125) Shou Z, Zhao Y, Zhang Y, Li S. Risk factors for peripheral arterial disease in elderly patients with Type-2 diabetes mellitus: a clinical study. Pak J Med Sci. 2020;36:1344-1348
126) Sigvant B, Wiberg-Hedman K, Bergqvist D, Rolandsson O, Wahlberg E. Risk factor profiles and use of cardiovascular drug prevention in women and men with peripheral arterial disease. Eur J Cardiovasc Prev Rehabil. 2009;16:39-46
127) Singh PP, Abbott JD, Lombardero MS, Sutton-Tyrrell K, Woodhead G, Venkitachalam L, et al. The prevalence and predictors of an abnormal ankle-brachial index in the Bypass Angioplasty Revascularization Investigation 2 Diabetes (BARI 2D) trial. Diabetes Care. 2011;34:464-467
128) Skalkidis Y, Katsouyanni K, Petridou E, Sehas M, Trichopoulos D. Risk factors of peripheral arterial occlusive disease: a case-control study in Greece. Int J Epidemiol. 1989;18:614-618
129) Smith FB, Connor JM, Lee AJ, Cooke A, Lowe GD, Rumley A, et al. Relationship of the platelet glycoprotein PlA and fibrinogen T/G+1689 polymorphisms with peripheral arterial disease and ischaemic heart disease. Thromb Res. 2003;112:209-216
130) Smith FB, Lee AJ, Rumley A, Fowkes FG, Lowe GD. Tissue-plasminogen activator, plasminogen activator inhibitor and risk of peripheral arterial disease. Atherosclerosis. 1995;115:35-43
131) Solanki JD, Makwana AH, Mehta HB, Gokhale PA, Shah CJ. A study of prevalence and association of risk factors for diabetic vasculopathy in an urban area of Gujarat. J Family Med Prim Care. 2013;2:360-364

132) Sticchi E, Sofi F, Romagnuolo I, Pratesi G, Pulli R, Pratesi C, et al. eNOS and ACE genes influence peripheral arterial disease predisposition in smokers. J Vasc Surg. 2010;52:97-102.e101

133) Stoekenbroek RM, Boekholdt SM, Luben R, Hovingh GK, Zwinderman AH, Wareham NJ, et al. Heterogeneous impact of classic atherosclerotic risk factors on different arterial territories: the EPIC-Norfolk prospective population study. Eur Heart J. 2016;37:880-889

134) Sumin AN, Bezdenezhnykh NA, Fedorova NV, Shcheglova AV, Indukaeva EV, Artamonova GV. The values of cardio-ankle vascular and ankle-brachial indices in patients with carbohydrate metabolic disorders: the ESSE-RF study in the Kemerovo Region. Ter Arkh. 2016;88:11-20

135) Syvänen K, Aarnio P, Jaatinen P, Korhonen P. Effects of age, sex and smoking on ankle-brachial index in a Finnish population at risk for cardiovascular disease. Int J Angiol. 2007;16:128-130

136) Tapp RJ, Balkau B, Shaw JE, Valensi P, Cailleau M, Eschwege E. Association of glucose metabolism, smoking and cardiovascular risk factors with incident peripheral arterial disease: the DESIR study. Atherosclerosis. 2007;190:84-89

137) Taylor-Piliae RE, Fair JM, Varady AN, Hlatky MA, Norton LC, Iribarren C, et al. Ankle brachial index screening in asymptomatic older adults. Am Heart J. 2011;161:979-985

138) Tellez-Plaza M, Guallar E, Fabsitz RR, Howard BV, Umans JG, Francesconi KA, et al. Cadmium exposure and incident peripheral arterial disease. Circ Cardiovasc Qual Outcomes. 2013;6:626-633

139) Thomas GN, Critchley JA, Tomlinson B, Cockram CS, Chan JC. Peripheral vascular disease in Type 2 diabetic Chinese patients: associations with metabolic indices, concomitant vascular disease and genetic factors. Diabet Med. 2003;20:988-995

140) Tmoyan NA, Ezhov MV, Afanasieva OI, Klesareva EA, Razova OA, Kukharchuk VV, et al. The association of lipoprotein(a) and apolipoprotein(a) phenotypes with peripheral artery disease. Ter Arkh. 2018;90:31-36

141) Tóth-Vajna G, Tóth-Vajna Z, Konkoly Thege B, Balog P. Depression among predictors of intermittent claudication: a cross-sectional study. Physiol Int. 2021

142) Tragante V, Doevendans PA, Nathoe HM, van der Graaf Y, Spiering W, Algra A, et al. The impact of susceptibility loci for coronary artery disease on other vascular domains and recurrence risk. Eur Heart J. 2013;34:2896-2904

143) Tseng CH. Prevalence and risk factors of peripheral arterial obstructive disease in Taiwanese type 2 diabetic patients. Angiology. 2003;54:331-338

144) Tyrrell J, Cooke T, Reilly M, Colgan M, Moore D, Shanik DG, et al. Lipoprotein [Lp(a)] and peripheral vascular disease. J Intern Med. 1992;232:349-352

145) Ungprasert P, Pornratanarangsi S. Correlation between peripheral arterial disease and stage of chronic kidney disease. J Med Assoc Thai. 2011;94 Suppl 1:S46-50

146) Unkart JT, Allison MA, Criqui MH, McDermott MM, Wood AC, Folsom AR, et al. Life's simple 7 and peripheral artery disease: the Multi-Ethnic Study of Atherosclerosis. Am J Prev Med. 2019;56:262-270
147) Urbano L, Portilla E, Muñoz W, Hofman A, Sierra-Torres CH. Prevalence and risk factors associated with peripheral arterial disease in an adult population from Colombia. Arch Cardiol Mex. 2018;88:107-115
148) Uusitupa MI, Niskanen LK, Siitonen O, Voutilainen E, Pyörälä K. 5-year incidence of atherosclerotic vascular disease in relation to general risk factors, insulin level, and abnormalities in lipoprotein composition in non-insulin-dependent diabetic and nondiabetic subjects. Circulation. 1990;82:27-36
149) Valdivielso P, Ariza MJ, de la Vega-Román C, González-Alegre T, Rioja J, Ulzurrun E, et al. Association of the -250G/A promoter polymorphism of the hepatic lipase gene with the risk of peripheral arterial disease in type 2 diabetic patients. J Diabetes Complications. 2008;22:273-277
150) Valentine RJ, Guerra R, Stephan P, Scoggins E, Clagett GP, Cohen J. Family history is a major determinant of subclinical peripheral arterial disease in young adults. J Vasc Surg. 2004;39:351-356
151) van Lennep HW, Westerveld HT, Zwinderman AH, van Lennep JE, Slot HB, Erkelens DW, et al. Differential effect of female gender on coronary artery disease and peripheral artery disease. Neth Heart J. 2002;10:500-505
152) Wang D, Zhang Q, Wang A, Wu S, Zhao X. Ideal Cardiovascular Health Metrics on the New Occurrence of Peripheral Artery Disease: A Prospective Cohort Study in Northern China. Sci Rep. 2020;10:9660
153) Wang HM, Chen TC, Jiang SQ, Liu YJ, Tian JW. Association of conventional risk factors for cardiovascular disease with IMT in middle-aged and elderly Chinese. Int J Cardiovasc Imaging. 2014;30:759-768
154) Wang X, Teng Q, Zhu L. Risk factors for development of PAD in PD patients. Iran J Kidney Dis. 2021;1:56-60
155) Wang Z, Wang X, Hao G, Chen Z, Zhang L, Shao L, et al. A national study of the prevalence and risk factors associated with peripheral arterial disease from China: the China Hypertension Survey, 2012-2015. Int J Cardiol. 2019;275:165-170
156) Wattanakit K, Folsom AR, Selvin E, Coresh J, Hirsch AT, Weatherley BD. Kidney function and risk of peripheral arterial disease: results from the Atherosclerosis Risk in Communities (ARIC) Study. J Am Soc Nephrol. 2007;18:629-636
157) Wattanakit K, Folsom AR, Selvin E, Weatherley BD, Pankow JS, Brancati FL, et al. Risk factors for peripheral arterial disease incidence in persons with diabetes: the Atherosclerosis Risk in Communities (ARIC) Study. Atherosclerosis. 2005;180:389-397
158) Weragoda J, Seneviratne R, Weerasinghe MC, Wijeyaratne M, Samaranayaka A. A cross-sectional study on peripheral arterial disease in a district of Sri Lanka: prevalence and associated factors. BMC Public Health. 2015;15:829
159) Wilson AM, Sadrzadeh-Rafie AH, Myers J, Assimes T, Nead KT, Higgins M, et al. Low lifetime recreational activity is a risk factor for peripheral arterial disease. J Vasc Surg. 2011;54:427-432, 432.e421-424

160) Woo J, Lynn H, Wong SY, Hong A, Tang YN, Lau WY, et al. Correlates for a low ankle-brachial index in elderly Chinese. Atherosclerosis. 2006;186:360-366

161) Yang X, Sun K, Zhang W, Wu H, Zhang H, Hui R. Prevalence of and risk factors for peripheral arterial disease in the patients with hypertension among Han Chinese. J Vasc Surg. 2007;46:296-302

162) Yuan S, Damrauer SM, Håkansson N, Åkesson A, Larsson SC. A prospective evaluation of modifiable lifestyle factors in relation to peripheral artery disease risk. Eur J Vasc Endovasc Surg. 2022

163) Yudkin JS, Forrest RD, Jackson CA. Microalbuminuria as predictor of vascular disease in non-diabetic subjects. Islington Diabetes Survey. Lancet. 1988;2:530-533

164) Zhang Y, Huang J, Wang P. A prediction model for the peripheral arterial disease using NHANES data. Medicine (Baltimore). 2016;95:e3454
