# Supplementary material for: Smoking as a risk factor for lower extremity peripheral artery disease in women compared to men: A systematic review and meta-analysis
Source: PLoS One. 2024 Apr 24;19(4):e0300963. doi: 10.1371/journal.pone.0300963 (PMC11042699; doi:10.1371/journal.pone.0300963)
Supplement: S3 File — (PDF) [file pone.0300963.s008.pdf]

S3 File List of included studies (n=17)

1. Banks E, Joshy G, Korda RJ, et al. Tobacco smoking and risk of 36 cardiovascular disease subtypes: fatal and non-fatal outcomes in a large prospective Australian study. *BMC Med*. 2019;17:128. doi: 10.1186/s12916-019-1351-4.

2. He Y, Jiang Y, Wang J, Fan L, Li X, Hu FB. Prevalence of peripheral arterial disease and its association with smoking in a population-based study in Beijing, China. *J Vasc Surg*. 2006;44:333-338. doi: 10.1016/j.jvs.2006.03.032.

3. Zheng L, Yu J, Li J, et al. Prevalence of and risk factors for peripheral arterial disease among Chinese hypertensive patients with and without known cardiovascular disease. *Acta Cardiol*. 2008;63:693-699. doi: 10.2143/ac.63.6.2033385.

4. Yi C, Junyi G, Fengju L, Qing Z, Jie C. Association between lipoprotein(a) and peripheral arterial disease in coronary artery bypass grafting patients. *Clin Cardiol*. 2023;46:512-520. doi: 10.1002/clc.24003.

5. Pujades-Rodriguez M, George J, Shah AD, et al. Heterogeneous associations between smoking and a wide range of initial presentations of cardiovascular disease in 1937360 people in England: lifetime risks and implications for risk prediction. *Int J Epidemiol*. 2015;44:129-141. doi: 10.1093/ije/dyu218.

6. Heikkilä A, Venermo M, Kautiainen H, Aarnio P, Korhonen P. Short stature in men is associated with subclinical peripheral arterial disease. *Vasa*. 2016;45:486-490. doi: 10.1024/0301-1526/a000566.

7. Jensen SA, Vatten LJ, Nilsen TI, Romundstad PR, Myhre HO. The association between smoking and the prevalence of intermittent claudication. *Vasc Med*. 2005;10:257-263. doi: 10.1191/1358863x05vm635oa.

8. Tunstall-Pedoe H, Peters SAE, Woodward M, Struthers AD, Belch JJF. Twenty-Year predictors of peripheral arterial disease compared with coronary heart disease in the Scottish Heart Health Extended Cohort (SHHEC). *J Am Heart Assoc*. 2017;6. doi: 10.1161/jaha.117.005967.

9. Ramos R, Quesada M, Solanas P, et al. Prevalence of symptomatic and asymptomatic peripheral arterial disease and the value of the ankle-brachial index to stratify cardiovascular risk. *Eur J Vasc Endovasc Surg*. 2009;38:305-311. doi: 10.1016/j.ejvs.2009.04.013.

10. Alzamora MT, Forés R, Baena-Díez JM, et al. The peripheral arterial disease study (PERART/ARTPER): prevalence and risk factors in the general population. *BMC Public Health*. 2010;10:38. doi: 10.1186/1471-2458-10-38.

11. Gonçalves-Martins G, Gil-Sala D, Tello-Díaz C, et al. Prevalence of peripheral arterial disease and associated vascular risk factors in 65-years-old people of Northern Barcelona. *J Clin Med*. 2021;10. doi: 10.3390/jcm10194467.

12. Bermúdez-López M, Martí-Antonio M, Castro-Boqué E, et al. Cumulative tobacco consumption has a dose-dependent effect on atheromatosis burden and improves severe atheromatosis prediction in asymptomatic middle-aged individuals: The ILERVAS study. *Atherosclerosis*. 2023;375:75-83. doi: 10.1016/j.atherosclerosis.2023.05.002.

13. Xu Y, Harris K, Pouncey AL, et al. Sex differences in risk factors for incident peripheral artery disease hospitalisation or death: cohort study of UK Biobank participants. *PLoS One*. 2023. doi: 10.1371/journal.pone.0292083.

14. Ness J, Aronow WS, Ahn C. Risk factors for symptomatic peripheral arterial disease in older persons in an academic hospital-based geriatrics practice. *J Am Geriatr Soc*. 2000;48:312-314. doi: 10.1111/j.1532-5415.2000.tb02652.x.

15. Lamar Welch VL, Casper M, Greenlund K, Zheng Z-J, Giles W, Rith-Najarian S. Prevalence of lower extremity arterial disease defined by the ankle-brachial index among American Indians: The inter-tribal heart project. *Ethn Dis*. 2002;12:S1.63-S61.67.

16. Zheng Z-J, Rosamond WD, Chambless LE, et al. Lower extremity arterial disease assessed by ankle-brachial index in a middle-aged population of African Americans and Whites: the Atherosclerosis Risk in Communities (ARIC) study. *Am J Prev Med*. 2005;29:42-49. doi: 10.1016/j.amepre.2005.07.019.

17. Hiramoto JS, Katz R, Weisman S, Conte M. Gender-specific risk factors for peripheral artery disease in a voluntary screening population. *J Am Heart Assoc*. 2014;3:e000651-e000651. doi: 10.1161/jaha.113.000651.
