# Supplementary material for: Smoking as a risk factor for lower extremity peripheral artery disease in women compared to men: A systematic review and meta-analysis
Source: PLoS One. 2024 Apr 24;19(4):e0300963. doi: 10.1371/journal.pone.0300963 (PMC11042699; doi:10.1371/journal.pone.0300963)
Supplement: Attachment — Submitted filename: Response to Reviewers.docx [file pone.0300963.s009.docx]

6 March 2024

The Academic Editor

The PLOS ONE

**RE:	Consideration of publication of our manuscript (PONE-D-23-43121): Smoking as a risk factor for lower extremity peripheral artery disease in women compared to men: a systematic review and meta-analysis**

Dear Professor Athanasios Saratzis,

We appreciate the opportunity to address the Reviewers’ comments and have revised our manuscript. Below, please find item-by-item responses to Reviewers’ comments.

Since it is required that any changes to the reference list should be mentioned in the rebuttal letter, we note that we added two publications in our reference list: 1) Jelani Q-u-a, Petrov M, Martinez SC, Holmvang L, Al-Shaibi K, Alasnag M. Peripheral arterial disease in women: an overview of risk factor profile, clinical features, and outcomes. Current Atherosclerosis Reports. 2018;20(8):40. doi: 10.1007/s11883-018-0742-x; and 2) Landis JR, Koch GG. The measurement of observer agreement for categorical data. Biometrics. 1977;33(1):159-174. doi: not available. In addition, we corrected an error that one reference was incorrectly listed twice: Xu Y, Harris K, Pouncey AL, Carcel C, Low G, Peters SAE, et al. Sex differences in risk factors for incident peripheral artery disease hospitalisation or death: cohort study of UK Biobank participants (embargo, to be published on 18 Oct 2023). PLoS One. 2023. doi: 10.1371/journal.pone.0292083.

Yours sincerely

Ying Xu

Research Fellow at Centre for Health Systems and Safety Research, Australian Institute of Health Innovation, Macquarie University

Honorary Research Fellow at The George Institute for Global Health

Reviewer #1: Thanks for the opportunity to review this manuscript reporting the results of a meta-analysis of studies reporting whether the relationship between smoking and peripheral arterial disease differs by sex. The authors have carried out a rigorous review of available literature and a statistically sound meta-analysis. Prior to publication, I recommend a few minor edits to be made:

Introduction/Discussion:

• Important to explain to the reader that there is significant under-recognition of cardiovascular risk in women and failure to recognise the importance of secondary prevention, sex-related differences in clinical presentation, and delay in presentation and misperception of cardiovascular disease in women. These factors contribute to peripheral arterial disease being underdiagnosed and understudied in women (see doi:10.1007/S11883-018-0742-X and PMID: 34010613). As well as in the introduction, it is worth emphasising this point in the discussion: Lines 313 – 326.

**Response:** Thank you for this good point. Other than the paragraph in Discussion, we have added more background for PAD in women in the Introduction, and now it reads “*The importance of cardiovascular risk in women is under-recognized, especially for peripheral artery disease (PAD). Due to higher rates of asymptomatic disease or atypical symptoms in women with PAD, they are often diagnosed at later stages of the disease, and thus are less likely to receive interventions to prevent advanced PAD and adverse outcomes of amputation, coronary heart disease (CHD), and stroke [1]*.” (lines 50 to 54)

Methodology:

• Line 82 - Authors mention passive smoking is not included yet they have included it in their search strategy

**Response:** This is a good point too. We did include “Passive Smoking” as a subject heading for one database CINAHL by mistake and it would unnecessarily increase the number of identified records and workload. We updated our search on 3 March 2024 and corrected this error.

In this search, we also removed “Limit S4 to Research or Journal Article, Human” in the CINAHL, because applying limits to “Research or Journal Article” did not reduce the number of identified records and the function of limit to “Human” was not available in this search.

No additional studies were added into our review from this updated search, but there were three additional records (listed below) that were excluded in the full-text screening process (S2 File), under “No reports on the association between smoking and peripheral arterial disease”:

Baretella O, Buser L, Andres C, Häberli D, Lenz A, Döring Y, et al. Association of sex and cardiovascular risk factors with atherosclerosis distribution pattern in lower extremity peripheral artery disease. Front Cardiovasc Med. 2023;10:1004003. doi: 10.3389/fcvm.2023.1004003.

Martelli E, Zamboni M, Sotgiu G, Saderi L, Federici M, Sangiorgi GM, et al. Sex-Related Differences and Factors Associated with Peri-Procedural and 1 Year Mortality in Chronic Limb-Threatening Ischemia Patients from the CLIMATE Italian Registry. J Pers Med. 2023;13(2). doi: 10.3390/jpm13020316.

Onofrei V, Adam CA, Marcu DTM, Leon MM, Cumpăt C, Mitu F, et al. Gender Differences and Amputation Risk in Peripheral Artery Disease-A Single-Center Experience. Diagnostics (Basel). 2023;13(19). doi: 10.3390/diagnostics13193145.

• Increased risk of bias given that only one reviewer has screened and identified articles – please discuss this in the limitations of the manuscript. (see: <https://doi.org/10.1016/j.jclinepi.2020.01.005>)

**Response:** We have now added this as a limitation (lines 348 to 351) “*First, the literature search, screening, and data extraction were conducted by one reviewer, although reference lists and citation trails were sourced to capture studies that may be missed out, and data extraction was checked multiple times by this reviewer and by a second reviewer*.”

• The authors state that they contacted individuals of potentially eligible studies. Please include how this was carried out and whether it was standardised.

“The authors of a few potentially eligible studies were contacted for their published full-text reports.”

**Response:** We added more details on how and whether standardised (lines 104 to 106). “*The authors of a few potentially eligible studies were contacted by emails with subjects/titles and contents drafted in a similar manner, for their published full-text reports.*”

As an example, here is an email to Professor Päivi Korhonen, email address: Arto.heikkila@fi mnet.fi

Dear Professor Päivi Korhonen,

Re Heikkilä A, Venermo M, Kautiainen H, Aarnio P, Korhonen P. Short stature in men is associated with subclinical peripheral arterial disease. Vasa. 2016 Nov;45(6):486-490. doi: 10.1024/0301-1526/a000566.

Please I may introduce myself. My name is Ying Xu. I am a Research Fellow at the George Institute for Global Health and Conjoint Lecture at the University of New South Wales in Sydney.

I am conducting a systematic review and meta-analysis on sex difference in smoking as a risk factor for peripheral artery disease.

I find the above-mentioned article of yours probably meet our inclusion criteria, but I cannot find the full text. May I please ask whether you would be happy to share the published full-text PDF or a word accepted version?

Many thanks,

Ying

Results:

• Line 149 & 150 – Please include SD of mean follow up & IQR of median follow up of the studies.

**Response:** we have now added the interquartile interval “(*interquartile interval 11.8, 13.3 years)*” (lines 160 to 161) for one study, but the numbers were not reported by other studies.

Reviewer #2: The authors submitted a systematic review and meta-analysis concerning smoking as risk factor for PAD in women related to men. The methodology is sound, and the conclusions justified. The discussion is weighty but addresses a variety of sex related differences in PAD. I think this paper excellent and is an important addition to the literature, complements on their work.

Thanks to authors for adhering to their Prospero submission. One major comment:

Line 100: The authors report only one reviewer screening all the documents by title, abstract and full text. This one reviewer also performed all the extractions with a second reviewer. Please account for this in your limitation section, as it introduces bias.

Minor comments:

Line 86: diagnostic and procedure codes, are these listed anywhere, can they be included?

**Response:** This is related to cohort studies, and we have now added the following text and point readers to S2b Table for codes “*Hospitalized or fatal PAD were identified using International Classification of Diseases and/or procedure codes in all cohort studies (S2b Table* *for codes) [14, 21-23]. One study used Health Data Research UK’s PAD phenotyping definitions and coding system and additionally identified PAD diagnoses in primary care [21].*” (lines 161 to 164) We also added “(search for “PH236” on <https://phenotypes.healthdatagateway.org/> )” in S2b Table for one study, England 2015, Pujades-Rodriguez, as a completed list would be lengthy.

Line 103: did you use a programme for citation review?

**Response:** We did not use a separate programme, although we have added more details on our citation review, which now it reads “*Each included study was identified on the Web of Science database, from where studies on the reference list and subsequent studies that cited it were exported to Endnote, followed by the same title, abstract, and full-text screening process.*” (lines 107 to 110)

Line 110: how much missing data did you encounter, is this reported in the results?

**Response:** In the Methods we now report that “*When relevant information was not reported in a study, we presented it as missing value.*”, and in the Results that ”*All extracted information (S2a-d Table, Figs 2 and 3) were found in the studies. There were some missing data in S2e Table, as the numbers of former, current, and/or never smokers among women, men, and/or the whole study sample were not reported in a few studies [15, 18, 22, 25-27, 29, 30].*” (lines 150 to 153)

Line 120: did you look at the level of agreement between your two reviewers? i.e. via cohen kappa.

**Response:** We have now added Cohen’s kappa along with some interpretations for reliability. In the Method, it reads “*Interrater reliability was measured by Cohen’s kappa.*” (line 127)

In the Results, it reads “*Cohen’s kappa was 0.42 for 28 items with 2 levels and 1 for 4 items with 3 levels in cohort studies. It was 0.46 for 52 items with 2 levels and 0.52 for 39 items with 3 levels in cross-sectional studies. After discussion, two reviewers reached agreements for all ratings in the quality assessments.*” (lines 175 to 178)

In the Discussion, “*Second, Cohen’s kappa between two reviewers for quality assessment was* *largely in the 0.4 to 0.59 range, indicating moderate level of agreement* [46]. (lines 351 to 353)

Line 143: may be worth stating this an upper-middle income country still.

Response: We made the sentence clearer as suggested, and it now reads “*Three studies were conducted in an upper-middle income country, China [27-29].*” (line 149-150)

Line 326: this sentence can strengthened with the result included within the sentence.

**Response:** We added the key finding to support this statement. Now, it reads “*Our results suggested at least the same value of smoking abstinence in women and men, considering the observed equal hazardous effects or inconsistencies in the direction of sex differences.*” (lines 342 to 344)
